# p66Shc mediates SUMO2-induced endothelial dysfunction

**DOI:** 10.7554/eLife.106344

**Published:** 2026-10-21

**Authors:** Jitendra Kumar, Shravan K Uppulapu, Sujata Kumari, Kanika Sharma, William J Paradee, Ravi Prakash Yadav, Vikas Kumar, Santosh Kumar

**Affiliations:** 1 Department of Internal Medicine, Division of Cardiovascular Medicine, University of Iowa Iowa City United States; 2 Department of Genetics, Cell Biology and Anatomy, University of Nebraska Medical Center Omaha United States; 3 Genome Editing Core Facility, University of Iowa Iowa City United States; 4 Regional Institute of Education-NCERT Mysore India; 5 Department of Biochemistry and Molecular Biotechnology, Mass Spectrometry Facility, University of Massachusetts Chan Medical School Worcester United States; Icahn School of Medicine at Mount Sinai United States; University of Cambridge United Kingdom

**Keywords:** endothelial function, p66Shc, sumoylation, Human, Mouse

## Abstract

Sumoylation is a post-translational modification that can regulate different physiological functions. Increased sumoylation, specifically conjugation of SUMO2/3 (small ubiquitin-like modifier 2/3), is detrimental to vascular health. However, the molecular mechanism mediating this effect is poorly understood. We used cell-based assays and mass spectrometry to show that p66Shc is a direct target of SUMO2 and that SUMO2 regulates p66Shc function via lysine-81 modification. To determine the effects of SUMO2-p66ShcK81 on vascular function, we generated Shc1*^K81R^* mice and crossbred them toLdlr*^-/-^* mice to induce hyperlipidemia. Next, to determine p66ShcK81-SUMO2ylation-induced changes in endothelial cell signaling, we performed mass spectrometry followed by Ingenuity Pathway Analysis. Our data reveal that p66Shc mediates the effects of SUMO2 on endothelial cells. Mass spectrometry identified that SUMO2 modified lysine-81 in the unique collagen homology-2 domain of p66Shc. SUMO2ylation of p66Shc increased phosphorylation at serine-36, causing it to translocate to the mitochondria, a step critical for the oxidative function of p66Shc. Notably, sumoylation-deficient p66Shc (p66ShcK81R) was resistant to SUMO2-induced p66ShcS36 phosphorylation and mitochondrial translocation. Shc1*^K81R^* mice were resistant to endothelial dysfunction induced by SUMO2ylation and hyperlipidemia. Ingenuity Pathway Analysis revealed multiple signaling pathways regulated by p66ShcK81-SUMO2ylation in endothelial cells, highlighting JAK-STAT as a major pathway affected by SUMO2-p66ShcK81. Collectively, our work reveals SUMO2-p66Shc signaling as a fundamental regulator of vascular endothelial function. We discovered that p66ShcK81 is an upstream modification regulating p66Shc signaling and mediates hyperlipidemia-induced endothelial dysfunction and oxidative stress.

## Introduction

Endothelial cells play an important role in regulating vascular health. One of the primary functions of endothelial cells is to maintain vascular tone and regulate blood flow in response to physiological changes. In the context of disease, endothelial cells either fail to produce enough nitric oxide (NO) or the available NO is quenched by reactive oxygen species (ROS). Consequently, this leads to endothelial dysfunction (ED), characterized by reduced availability of NO and impaired vasorelaxation (Ludmer et al., 1986). ED is associated with various conditions, such as diabetes, hyperlipidemia, hyperglycemia, obesity, and aging (Lundberg and Weitzberg, 2022). There is a strong correlation between ED and atherosclerotic changes in vessel walls (Dai et al., 2004). Therefore, ED is one of the prime targets to prevent pathologies, including myocardial infarction and stroke.

Sumoylation is a dynamic post-translational modification involved in many pathologies, including vascular disease (Chang and Yeh, 2020). Increased sumoylation promotes ED and subsequent development of atherosclerosis (Heo et al., 2015; Heo et al., 2013). Sumoylation occurs by conjugation of **s**mall **u**biquitin-like **mo**difier**s** (SUMOs) to the lysine moiety of the target protein, which may change its fate and/or function. SUMO conjugation is predicted to occur at a specific motif, ΨKXE, where Ψ represents an aliphatic-branched amino acid, X a random amino acid, and E glutamic acid, although other non-canonical sumoylation sites have also been reported (Chang and Yeh, 2020; Song et al., 2004; Impens et al., 2014). There are four SUMO isoforms (SUMO1, SUMO2, SUMO3, and SUMO4), and among them SUMO2 is the most abundantly expressed. Unlike SUMO1 and SUMO3, genetic deletion of SUMO2 is embryonically lethal (Wang et al., 2014; Zhang et al., 2008). While sumoylation is known to promote cardiovascular diseases, the effect of SUMO isoforms on the vasculature is not well studied. Recently, we reported that endothelial-specific overexpression of SUMO2 impairs vascular endothelial function in mice (Kim et al., 2019). However, the molecular mechanisms mediating the endothelial effects of SUMO2 are not known.

p66Shc is a member of the ShcA family of adaptor proteins. Among the ShcA protein family (p66Shc, p52Shc, and p46Shc), p66Shc is the largest protein due to an additional collagen homology domain (CH2) at its N-terminus. Posttranslational modifications in the CH2 domain regulate the oxidative function of p66Shc (Pinton et al., 2007; Khanday et al., 2006; Kumar et al., 2017; Pacini et al., 2004), whereas phosphorylation in the CH1 domain leads to mitogenic signaling (Rozakis-Adcock et al., 1992; Gotoh et al., 1996) a period p66Shc is abundantly expressed in endothelium and mediates ED due to hyperlipidemia, hyperglycemia, and aging (Kumar, 2019). Correspondingly, mice with a genetic deletion of p66Shc have a longer lifespan and are protected against ED due to metabolic disorders (Napoli et al., 2003; Camici et al., 2007). Here, we examined whether p66Shc mediates the effects of SUMO2 on endothelial cells. Using multiple techniques, we show that SUMO2 directly modifies p66Shc at lysine-81 in its CH2 domain, which is essential for SUMO2-induced ROS production and ED. Thus, our study uncovers a previously unknown posttranslational modification that regulates p66Shc function in the endothelium and defines a novel molecular mechanism by which increased SUMO2ylation induces ROS, leading to vascular ED.

## Results

### p66Shc mediates SUMO2-induced ROS production in endothelial cells

**Figure 1.**
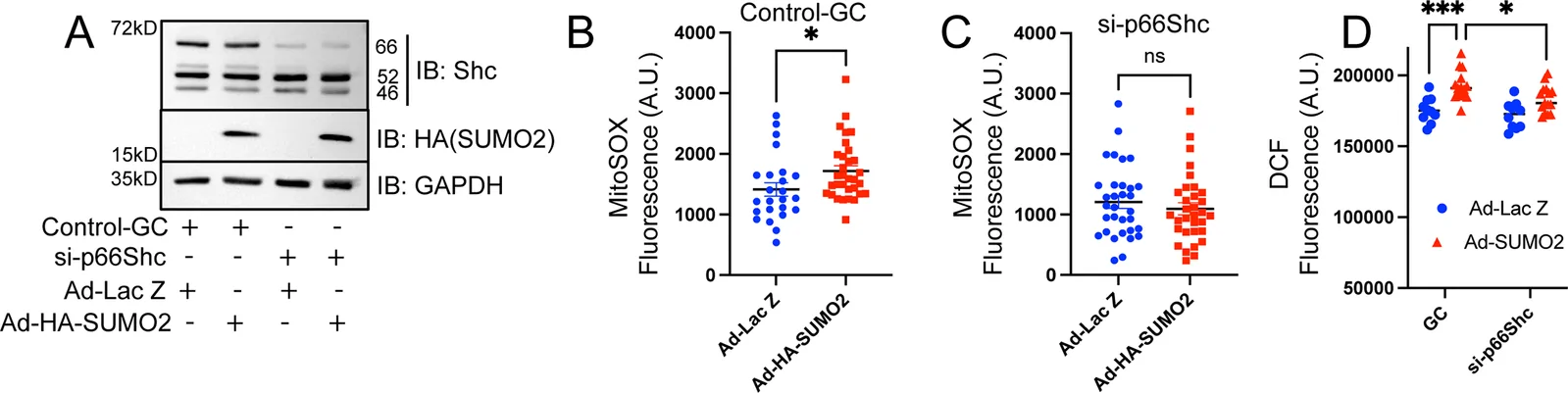
p66Shc mediates small ubiquitin-like modifier 2 (SUMO2)-induced reactive oxygen species (ROS) production and inflammation in endothelial cells. (**A**) Immunoblot for Shc and SUMO2 expression in HUVECs following p66Shc knockdown (si-p66Shc) and SUMO2 overexpression (Ad-HA-SUMO2). (**B, C**) Quantification of ROS (MitoSOX fluorescence) in HUVECs from A. *p<0.05; n=24–32; Student’s *t*-test. (**D**) Quantification of ROS in HUVECs following p66Shc knockdown (si-p66Shc) and SUMO2 overexpression (Ad-HA-SUMO2). ***p<0.001, p<0.05; n=10–15; One-way ANOVA. Data represents mean ± SEM. Immunoblots are representative of at least three independent experiments. GC, scrambled siRNA used as control; HUVECs, human umbilical vein endothelial cells. Figure 1—source data 1.PDF file containing original western blots for Figure 1. Figure 1—source data 2.Original files for western blot analysis displayed in Figure 1.

To address whether p66Shc mediates SUMO2-induced ROS production, we overexpressed SUMO2 in human umbilical vein endothelial cells (HUVECs) with and without knockdown of p66Shc and used MitoSOX to detect mitochondrial levels of ROS, a prominent function of p66Shc. As expected, overexpression of SUMO2 led to a robust increase in levels of ROS (Figure 1A and B). This SUMO2-induced increase in ROS was significantly blunted in HUVECs with p66Shc knockdown (Figure 1A and C). In a complementary approach, we used H_2_DCFDA to determine cellular ROS levels and noted a similar increase in ROS levels with overexpression of SUMO2 which was significantly reduced by the knockdown of p66Shc (Figure 1D). This suggests that SUMO2 is targeting p66Shc for ROS production in endothelial cells.

### p66Shc is directly modified by SUMO2

**Figure 2.**
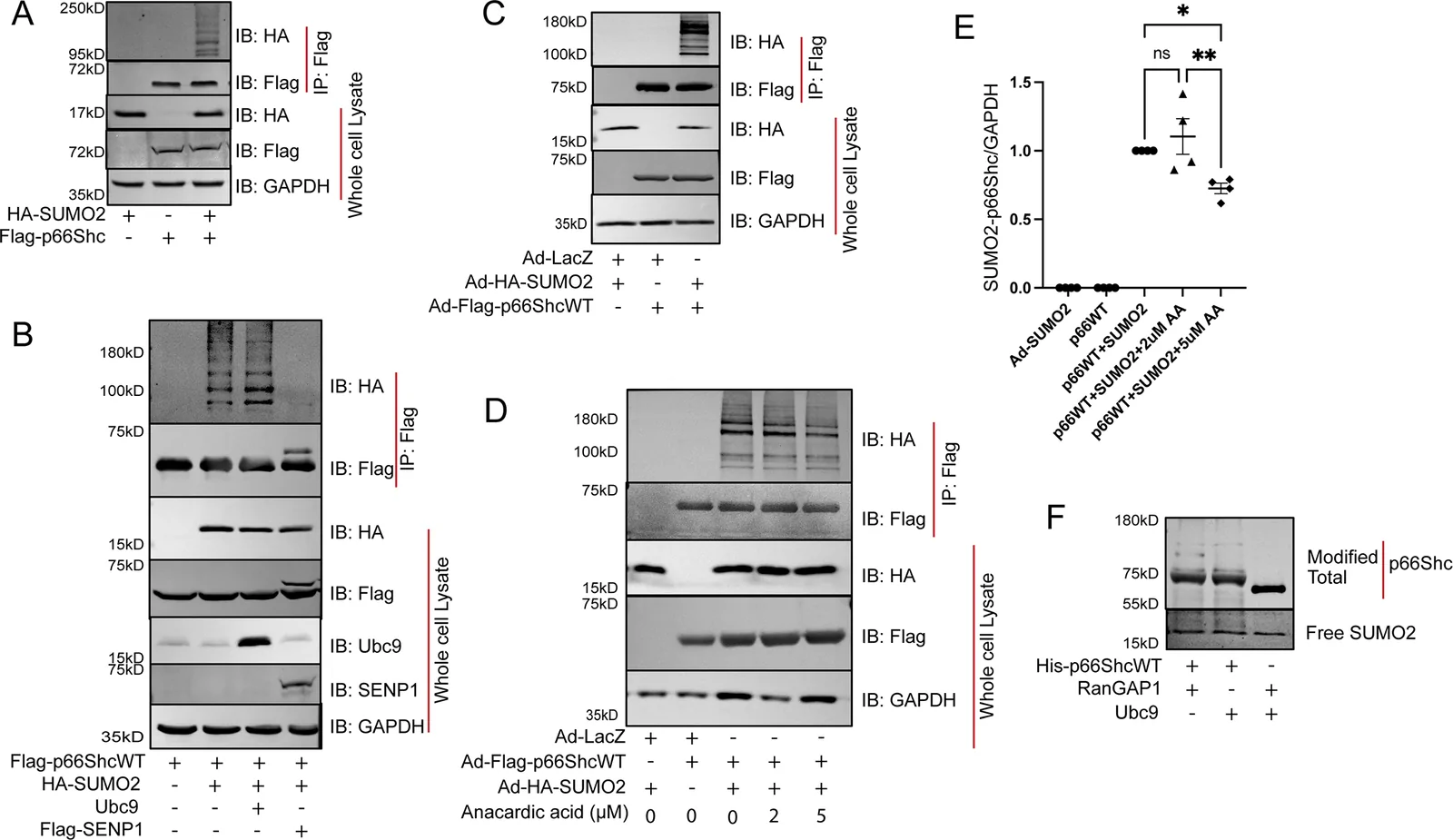
Small ubiquitin-like modifier 2 (SUMO2) directly modifies p66Shc. (**A**) Immunoblot for sumoylated p66Shc in HEK-293 cells overexpressing SUMO2 (HA-SUMO2) and p66Shc (Flag-p66Shc). (**B**) Immunoblot for p66Shc sumoylation in HEK-293 cells overexpressing SUMO2 (HA-SUMO2) and p66Shc (Flag-p66Shc) with or without overexpression of Ubc9 (HA-Ubc9) and SENP1 (Flag-SENP1). (**C**) Immunoblot for sumoylation of p66Shc in human umbilical vein endothelial cells (HUVECs) overexpressing SUMO2 (Ad-HA-SUMO2) or p66Shc (Ad-Flag-p66Shc). (**D**) Immunoblot for p66Shc sumoylation in HUVEC cells overexpressing SUMO2 (Ad-HA-SUMO2) or p66Shc (Ad-Flag-p66Shc) in the presence or absence of anacardic acid. Quantification of SUMO2-p66Shc from immunoblot of D. One-way ANOVA, followed by Tukey’s post-hoc analysis, n=4 *p<0.05, **p<0.01. (**F**) Immunoblot for sumoylated recombinant p66Shc in the presence of absence of Ubc9. Immunoblots are representative of at least three independent experiments. Data represents mean ± SEM. Ad-Lac Z was used as an adenoviral control to compensate for the difference in MOI among the groups. Figure 2—source data 1.PDF file containing original western blots for Figure 2. Figure 2—source data 2.Original files for western blot analysis displayed in Figure 2.

We next asked whether p66Shc is a direct target for SUMO2. We overexpressed p66Shc in HEK-293 cells with and without SUMO2. Immunoprecipitation of p66Shc showed many slower migrating protein bands when SUMO2 and p66Shc were co-expressed, suggesting that p66Shc may be SUMO2ylated (Figure 2A). To confirm that the slower migrating bands were due to sumoylation, we overexpressed SUMO-ligase (Ubc9) to facilitate sumoylation, and a deSUMOylating enzyme (SENP1) which removes SUMO proteins. Overexpression of Ubc9 increased the signal of slower migrating bands, whereas SENP1 reduced the signal suggesting that the slower migrating bands were due to sumoylation (Figure 2B). Next, we asked whether the SUMO2-p66Shc interaction exists in endothelial cells by overexpressing SUMO2 and p66Shc in HUVECs. Immunoprecipitation of Flag-tagged p66Shc revealed sumoylation (Figure 2C), which consistently increased in intensity with increasing SUMO2 expression (Figure 2—figure supplement 1). To verify that p66Shc undergoes SUMO2ylation in HUVECs, we treated cells with a pharmacological inhibitor of sumoylation (anacardic acid). This reduced sumoylation of p66Shc (Figure 2D and E), demonstrating that indeed p66Shc undergoes SUMO2ylation in endothelial cells while the levels of total p66Shc remain unchanged (Figure 2—figure supplement 2A and B). We also performed an in vitro sumoylation of p66Shc using recombinant His-tagged p66Shc protein and a commercially available kit (Enzo, Farmingdale, NY), which showed that recombinant p66Shc is modified by SUMO2 (Figure 2F). Collectively, these findings indicate that p66Shc is a direct target of SUMO2.

**Figure 2—figure supplement 1.**
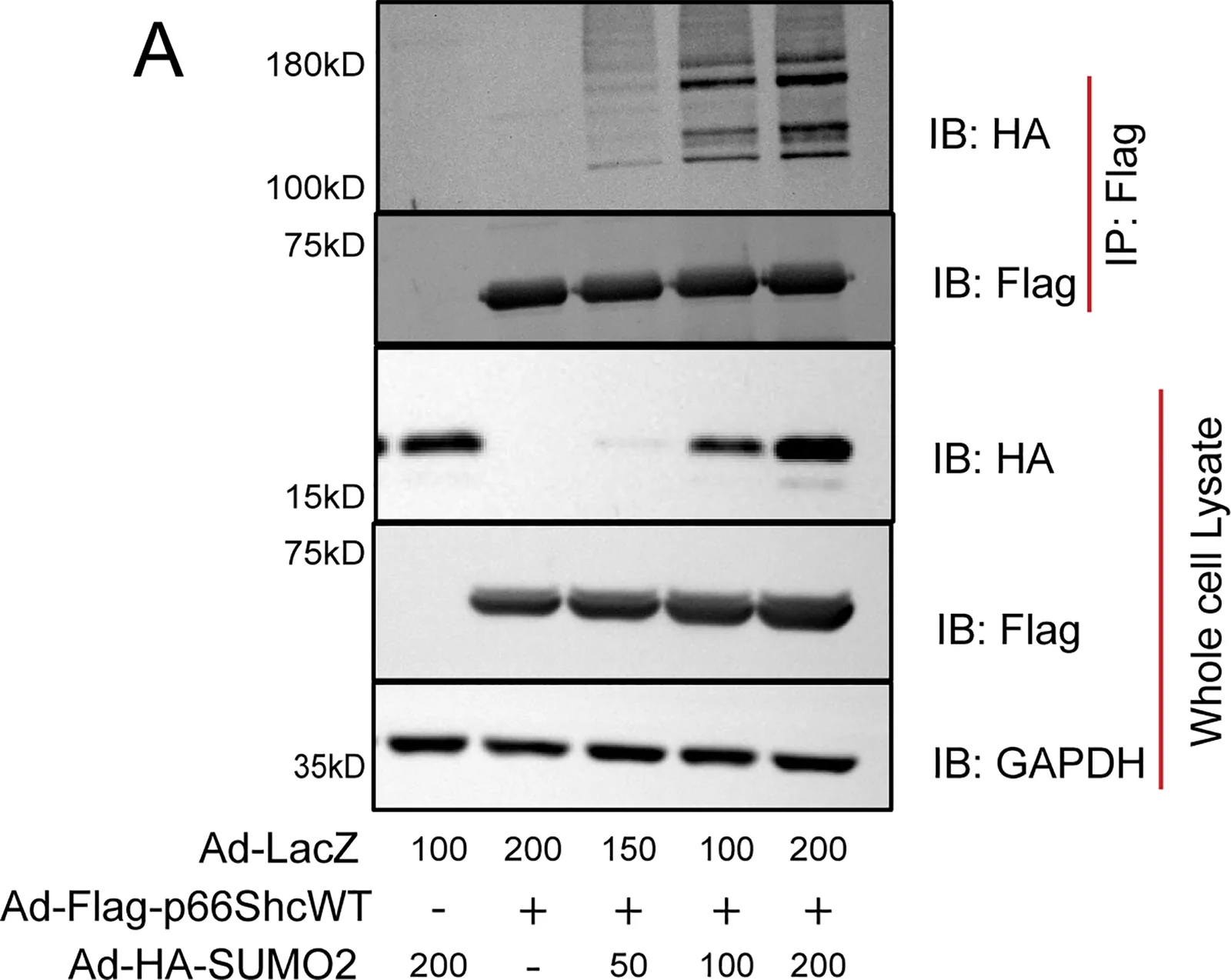
Small ubiquitin-like modifier 2 (SUMO2) promotes p66Shc SUMO2ylation in a dose-dependent manner. Immunoblot of human umbilical vein endothelial cells (HUVECs) transduced with wild-type p66Shc (Ad-Flag-p66ShcWT) and SUMO2 (Ad-HA-SUMO2) at the indicated concentrations. Immunoblots are representative of at least three independent experiments. Figure 2—figure supplement 1—source data 1.PDF file containing original western blots for Figure 2—figure supplement 1. Figure 2—figure supplement 1—source data 2.Original files for western blot analysis displayed in Figure 2—figure supplement 1.

**Figure 2—figure supplement 2.**
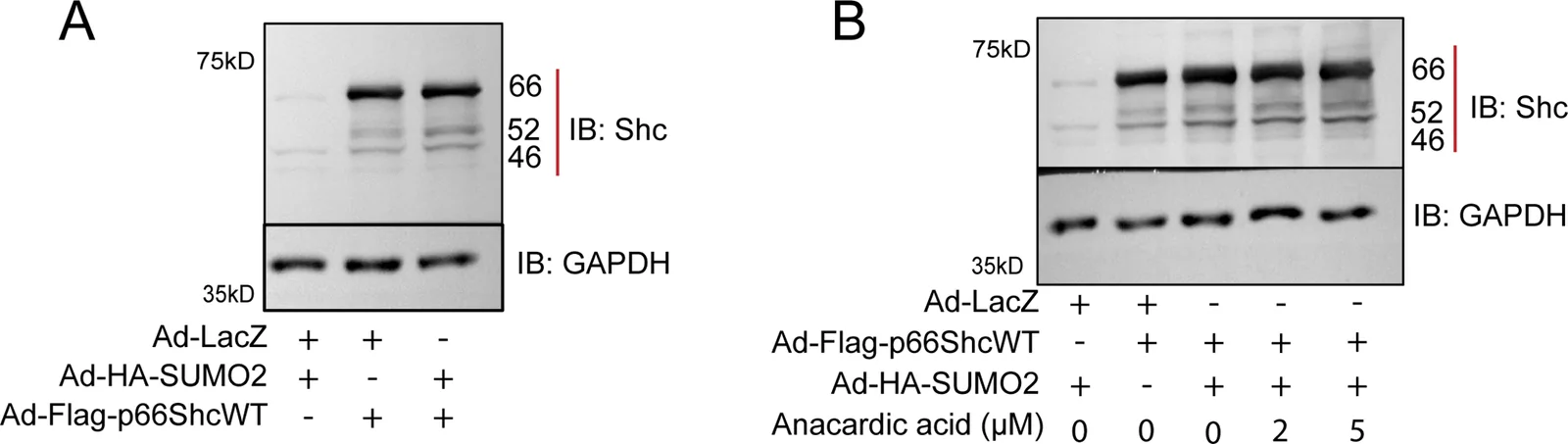
Immunoblot showing the relative overexpression level of p66Shc in human umbilical vein endothelial cells (HUVECs) used for (**A**) experiment 2 C and (**B**) experiment 2D. Figure 2—figure supplement 2—source data 1.PDF file containing original western blots for Figure 2—figure supplement 2A. Figure 2—figure supplement 2—source data 2.Original files for western blot analysis displayed in Figure 2—figure supplement 2A. Figure 2—figure supplement 2—source data 3.PDF file containing original western blots for Figure 2—figure supplement 2B. Figure 2—figure supplement 2—source data 4.Original files for western blot analysis displayed in Figure 2—figure supplement 2B.

### SUMO2 modifies p66Shc via lysine-81

**Figure 3.**
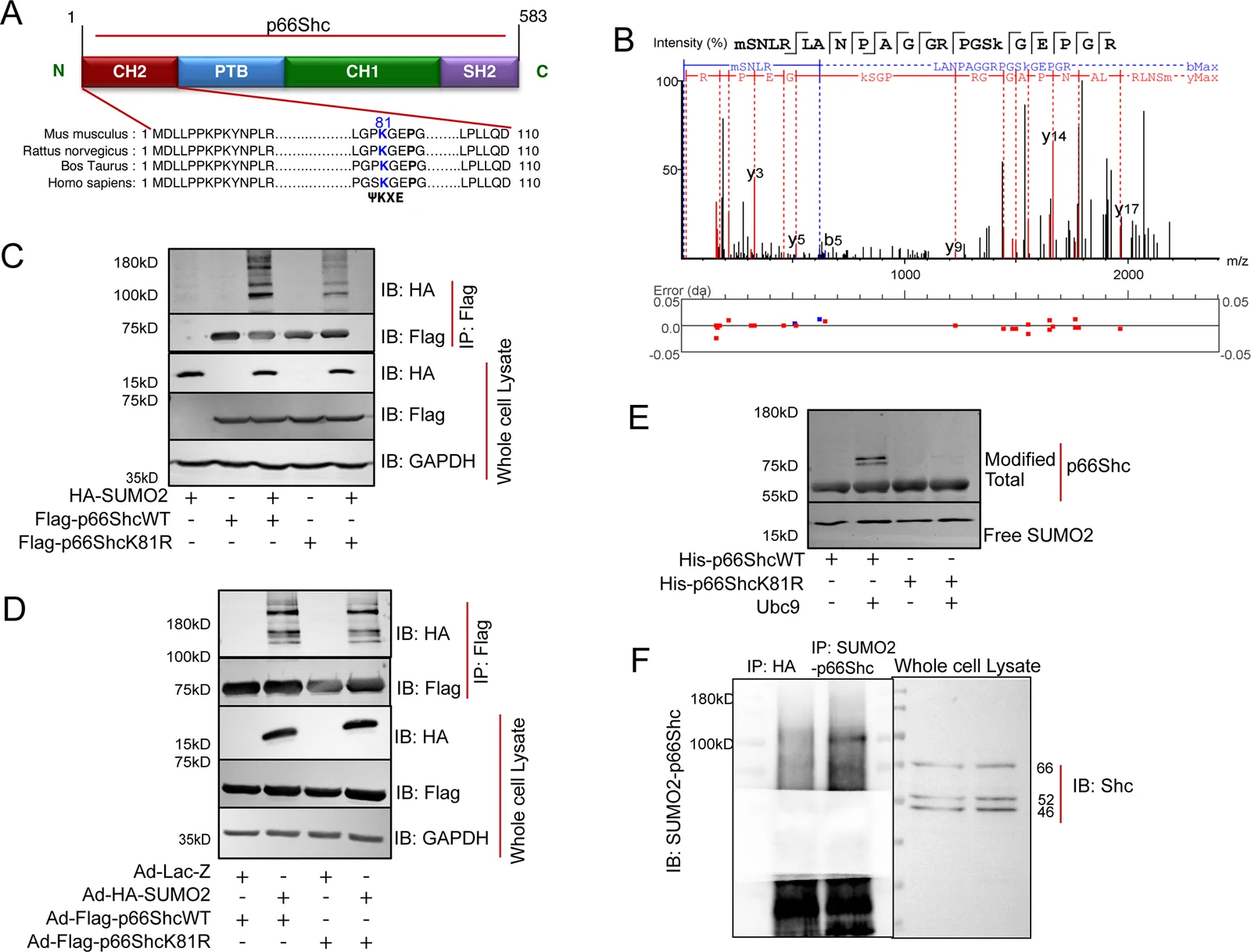
Small ubiquitin-like modifier 2 (SUMO2) modifies p66Shc at lysine-81. (**A**) Modular structure of p66Shc showing conserved lysine-81 in the CH2 domain. (**B**) Tandem mass spectrometry of in vitro SUMO2ylated recombinant p66Shc. (**C**) Immunoblot showing sumoylation of p66Shc in HEK-293 cells overexpressing p66ShcWT (Flag-p66ShcWT) or p66ShcK81R (Flag-p66ShcK81R) with and without SUMO2 (HA-SUMO2). (**D**) Immunoblot showing sumoylation of p66Shc in human umbilical vein endothelial cells (HUVECs) overexpressing p66ShcWT (Ad-Flag-p66ShcWT) or p66ShcK81R (Ad-p66ShcK81R) with and without SUMO2 (Ad-HA-SUMO2). (**E**) Immunoblot for sumoylation of recombinant p66ShcWT or p66ShcK81R. (**F**) Immunoprecipitation of SUMO2-p66Shc using SUMO2-p66ShcK81 antibody or the matching IgG control in HUVECs followed by immunoblotting with SUMO2-p66ShcK81 antibody showing endogenous SUMO2ylation of p66Shc (left-side panel, the high-intensity IgG signal was masked with a tape which appears as a white strip). Immunoblotting with Shc antibody in whole-cell lysate (right-side panel). Immunoblots are representative of at least three independent experiments. CH2, collagen homology 2; PTB, phosphotyrosine binding; CH1, collagen homology 1; SH2, Src homology 2. Figure 3—source data 1.PDF file containing original western blots for Figure 3C. Figure 3—source data 2.Original files for western blot analysis displayed in Figure 3C. Figure 3—source data 3.PDF file containing original western blots for Figure 3D. Figure 3—source data 4.Original files for western blot analysis displayed in Figure 3D. Figure 3—source data 5.PDF file containing original western blots for Figure 3E. Figure 3—source data 6.Original files for western blot analysis displayed in Figure 3E. Figure 3—source data 7.PDF file containing original western blots for Figure 3F. Figure 3—source data 8.Original files for western blot analysis displayed in Figure 3F.

The CH2 domain of p66Shc is highly conserved and involved in the oxidative function of p66Shc. Within this region, there are several conserved lysine residues. Among these is lysine 81, which is part of the ΨKXE motif required for sumoylation (Figure 3A). Therefore, to examine if K81 is sumoylated, we took advantage of SUMO2ylated recombinant p66Shc and resolved it on SDS-PAGE. Based on the approximate location of modified protein bands seen in immunoblotting, we cut out the gel piece and subjected it to mass spectrometry analysis (Figure 3—figure supplement 1). This demonstrated that lysine-81 (K81) in p66Shc was SUMO2ylated, identified as lysine conjugated with the QTGG motif present at the C-terminus of SUMO2 (Figure 3B). To confirm that SUMO2 modifies p66Shc at K81, we mutated this residue to arginine (p66ShcK81R) and overexpressed it in HEK-293 cells with SUMO2 to determine if sumoylation still occurred. Immunoprecipitation showed that p66ShcK81R is resistant to SUMO2ylation, whereas it still occurred with the wild-type (WT) form (Figure 3C). Similar results were also observed in HUVECs (Figure 3D). We next performed an in vitro SUMO2ylation of recombinant p66ShcWT and p66ShcK81R. As anticipated, immunoblotting showed that, unlike p66ShcWT, p66ShcK81R is resistant to SUMO2ylation (Figure 3E). Next, to determine the endogenous SUMO2-p66Shc, we generated a custom antibody against the branched peptide, mimicking the region of SUMO2 attachment (SUMO2-K81) to p66Shc using a commercial source (YenZym, CA). Using this SUMO2-p66ShcK81 antibody, we performed immunoprecipitation and immunoblotting in HUVECs treated with o-LDL (200 μg/ml) for 48 hr and noted p66Shc-SUMO2ylation at endogenous level (Figure 3F). Thus, our findings demonstrate that SUMO2 modifies p66Shc at K81.

**Figure 3—figure supplement 1.**
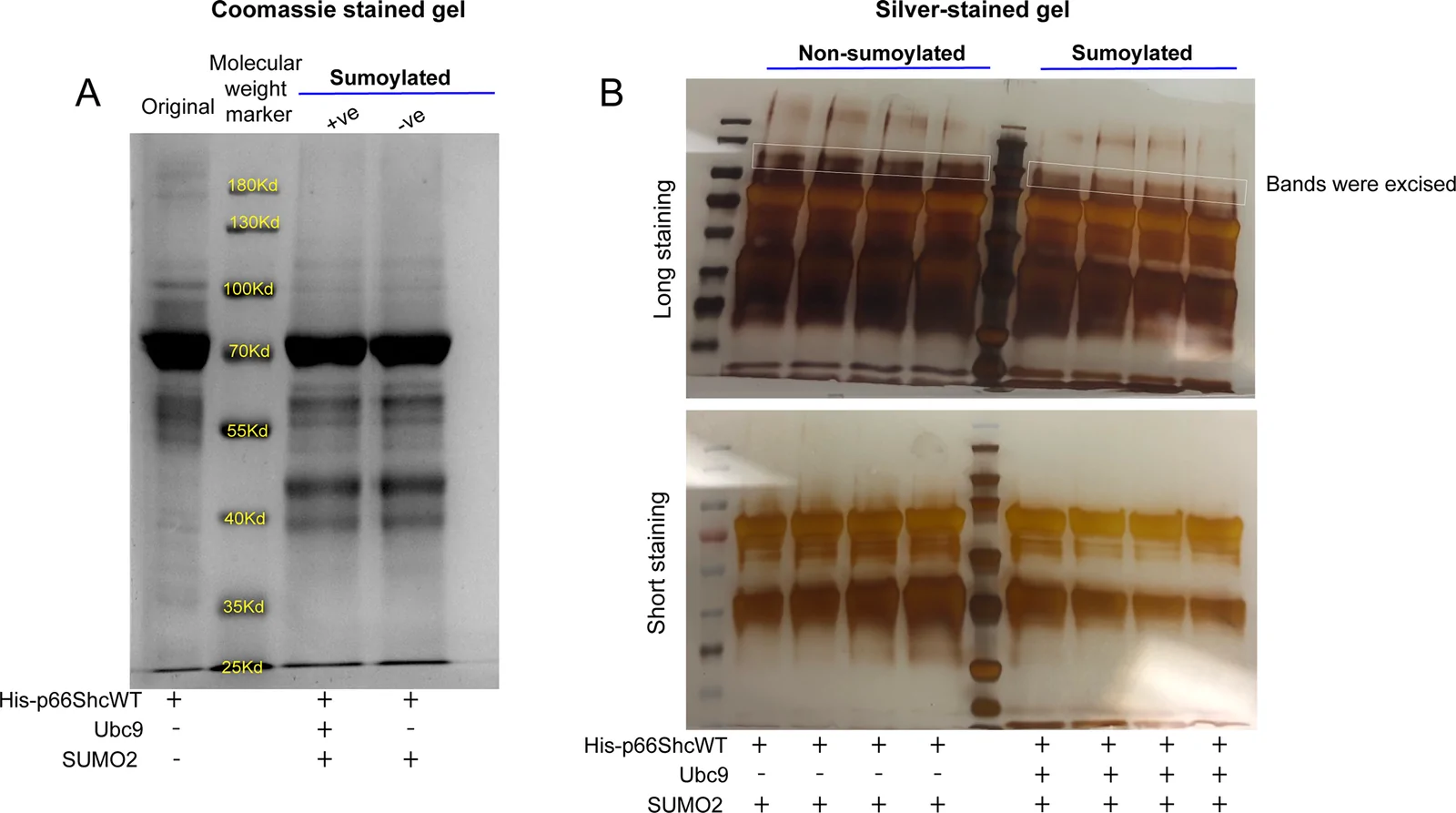
Sumoylation of recombinant p66ShcWT for mass spectrometry. (**A**) Coomassie stain of SDS-PAGE of recombinant p66ShcWT in its original form (not processed for SUMO2ylation), and after being SUMO2ylated and non-SUMO2ylated. (**B**) Silver stain of non-SUMO2ylated and SUMO2ylated recombinant p66ShcWT used for mass spectrometry. Figure 3—figure supplement 1—source data 1.PDF file containing original western blots for Figure 3—figure supplement 1A. Figure 3—figure supplement 1—source data 2.Original files for western blot analysis displayed in Figure 3—figure supplement 1A.

### SUMO2 promotes serine 36 phosphorylation and mitochondrial translocation of p66Shc

**Figure 4.**
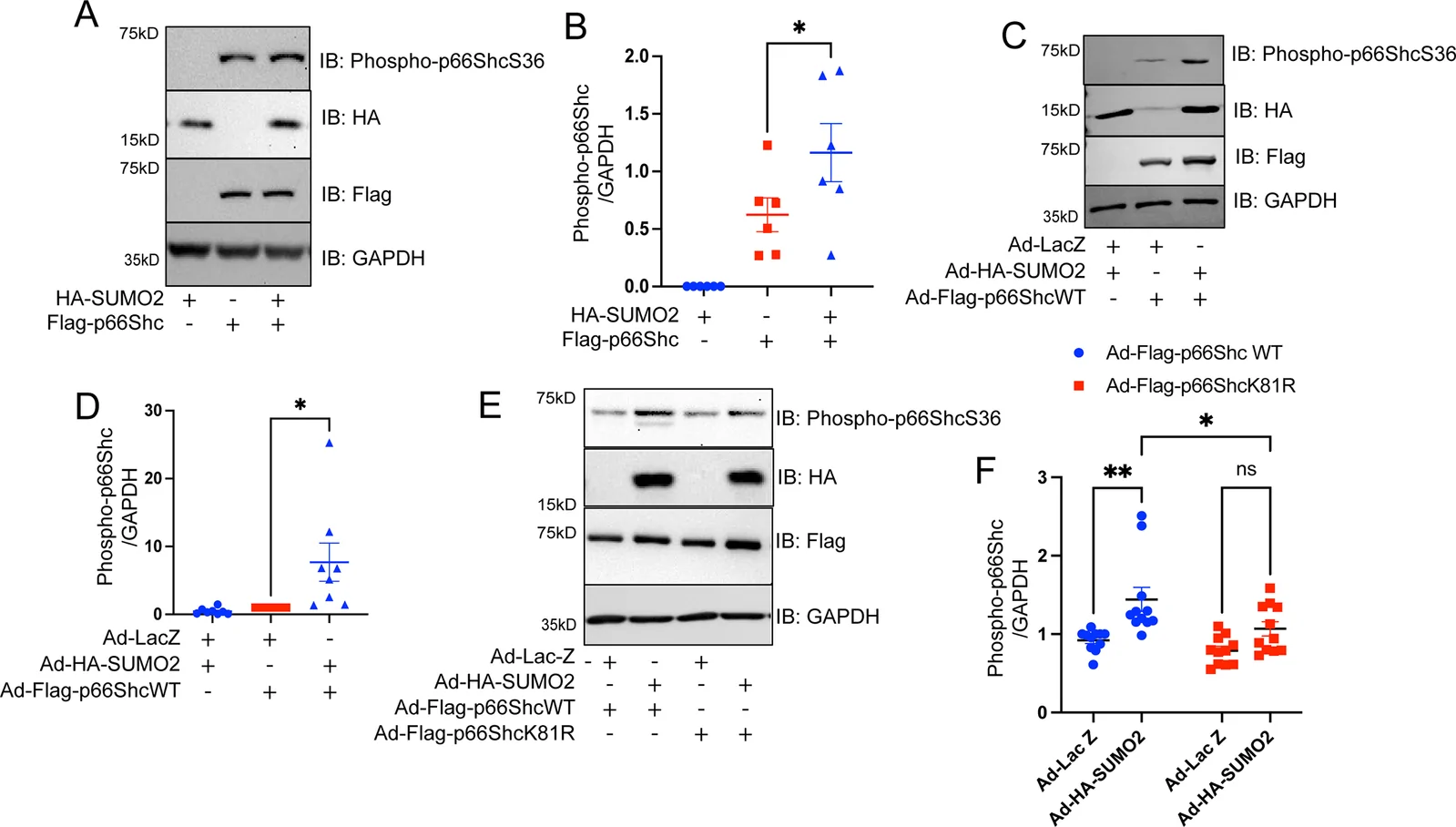
Small ubiquitin-like modifier 2 (SUMO2) promotes p66ShcS36 phosphorylation by sumoylation of K81. (**A**) Immunoblot for p66ShcS36 phosphorylation in HEK-293 cells overexpressing SUMO2 (HA-SUMO2) and p66ShcWT (Flag-p66ShcWT). (**B**) Quantification of p66ShcS36 phosphorylation from A. *p<0.05; n=6; one-way ANOVA. (**C**) Immunoblot for p66ShcS36 phosphorylation in human umbilical vein endothelial cells (HUVECs) overexpressing SUMO2 (Ad-HA-SUMO2) or p66ShcWT (Ad-Flag-p66ShcWT). (**D**) Quantification of p66ShcS36 phosphorylation from C. *p<0.05; n=8; one-way ANOVA. (**E**) Immunoblot for p66ShcS36 phosphorylation in HUVECs expressing SUMO2 (Ad-HA-SUMO2) and p66ShcWT (Ad-Flag-p66ShcWT) or p66ShcK81R (Ad-Flag-p66ShcK81R). (**F**) Quantification of p66ShcS36 phosphorylation from E. *p<0.05, **p<0.01; n=11; two-way ANOVA. Immunoblots were quantified with iBright image analysis software. Data represents mean ± SEM. Immunoblots are representative of at least three independent experiments. Figure 4—source data 1.PDF file containing original western blots for Figure 4A. Figure 4—source data 2.Original files for western blot analysis displayed in Figure 4A. Figure 4—source data 3.PDF file containing original western blots for Figure 4C. Figure 4—source data 4.Original files for western blot analysis displayed in Figure 4C. Figure 4—source data 5.PDF file containing original western blots for Figure 4E. Figure 4—source data 6.Original files for western blot analysis displayed in Figure 4E.

**Figure 5.**
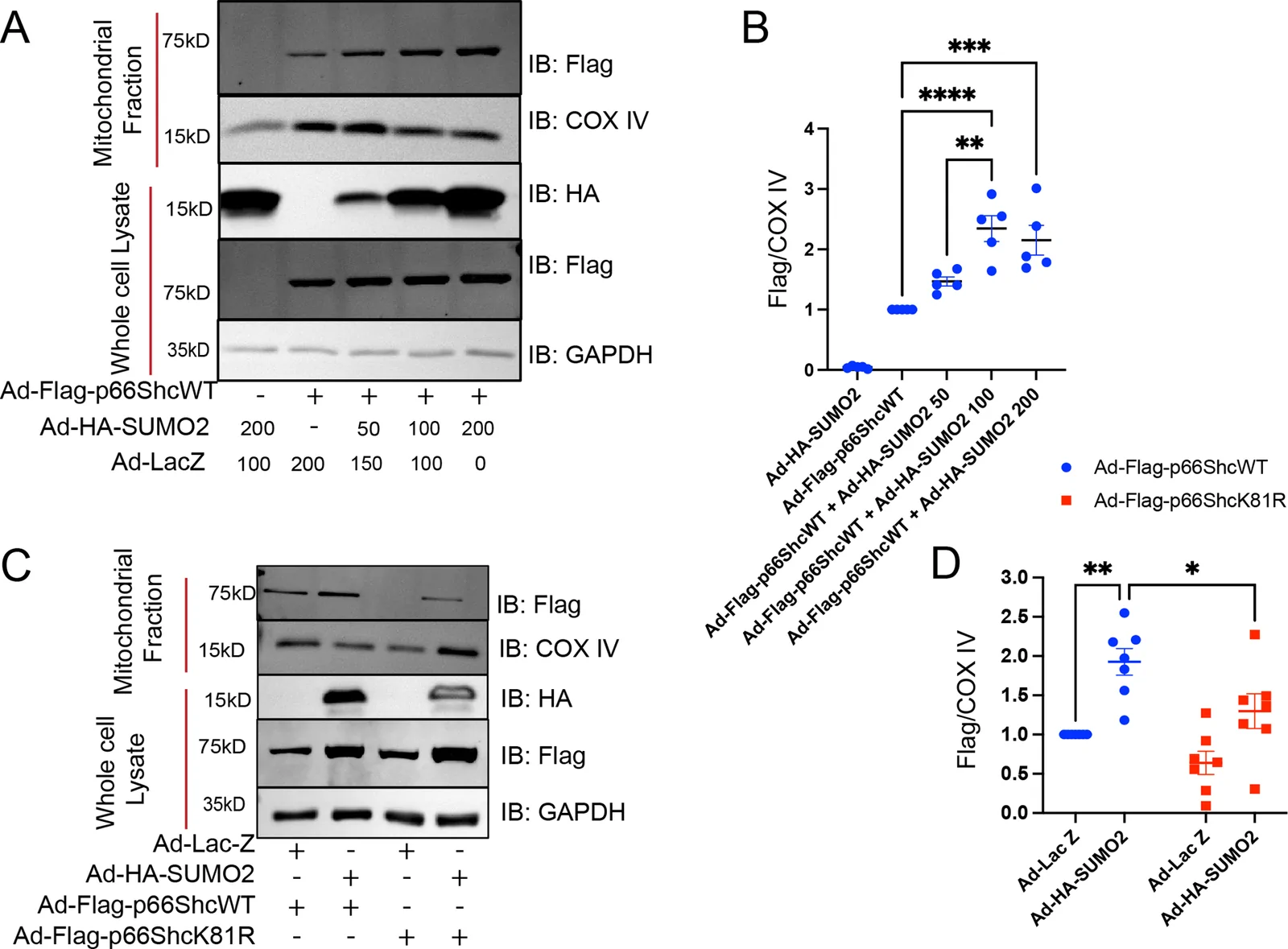
p66ShcK81 SUMO2ylation of K81 in p66Shc promotes its translocation to the mitochondria. (**A**) Immunoblot for p66ShcWT in whole-cell lysates or the mitochondrial fraction of human umbilical vein endothelial cells (HUVECs) ectopically expressing SUMO2 (Ad-HA-SUMO2) and p66ShcWT (Ad-Flag-p66ShcWT). (**B**) Quantification of the relative abundance of p66Shc in the mitochondria from A. **p<0.01, ****p<0.0001; n=5; one-way ANOVA. (**C**) Immunoblot for p66Shc in whole-cell lysates or the mitochondrial fraction of HUVECs ectopically expressing SUMO2 (Ad-HA-SUMO2) and p66ShcWT Ad-Flag-p66ShcWT or p66ShcK81R (Ad-Flag-p66ShcK81R). (**D**) Quantification of relative abundance of p66Shc in the mitochondria from C. *p<0.05, **p<0.01; n=7; two-way ANOVA. Data represents mean ± SEM. Immunoblots are representative of at least three independent experiments. Figure 5—source data 1.PDF file containing original western blots for Figure 5A. Figure 5—source data 2.PDF file containing original western blots for Figure 5A. Figure 5—source data 3.PDF file containing original western blots for Figure 5C. Figure 5—source data 4.Original files for western blot analysis displayed in Figure 5C.

In response to pro-oxidant stimuli, p66Shc becomes phosphorylated at serine 36 (S36) and translocates to mitochondria. Once there, it oxidizes cytochrome c to produce ROS (Wright et al., 2018; Giorgio et al., 2005), which is a central mechanism to activate the oxidative function of p66Shc. Since, the K81 and S36 residues of p66Shc are both present in its conserved CH2 domain, we asked whether promoting SUMO2ylation also facilitates phosphorylation of the S36 residue of p66Shc. In HEK-293 cells as well as in HUVECs, we noted increased levels of p66ShcS36 phosphorylation when SUMO2 and p66Shc were co-expressed (Figure 4A–D). To determine whether SUMO2-induced S36 phosphorylation is mediated via K81 sumoylation, we evaluated p66ShcS36 phosphorylation in HUVECs overexpressing p66ShcK81R and SUMO2. SUMO2 overexpression significantly increased S36 phosphorylation in cells expressing p66ShcWT, whereas this response was markedly attenuated in cells expressing p66ShcK81R (Figure 4E and F), indicating that K81 SUMOylation promotes, but is not strictly required for, S36 phosphorylation. As p66ShcS36 phosphorylation increases p66Shc levels in mitochondria, we evaluated whether SUMO2 affects mitochondrial levels of p66Shc. Levels of p66Shc in the mitochondria of HUVECs increased in a dose-dependent manner upon SUMO2 overexpression (Figure 5A and B). However, mitochondrial expression of p66Shc was significantly lower in HUVECs expressing p66ShcK81R relative to p66ShcWT (Figure 5C and D). Collectively, these findings support a model in which SUMO2ylation at K81 facilitates S36 phosphorylation and enhances mitochondrial translocation of p66Shc, thereby promoting its oxidative signaling function.

### Mice expressing non-sumoylatable p66ShcK81R are protected against SUMO2-induced ED

**Figure 6.**
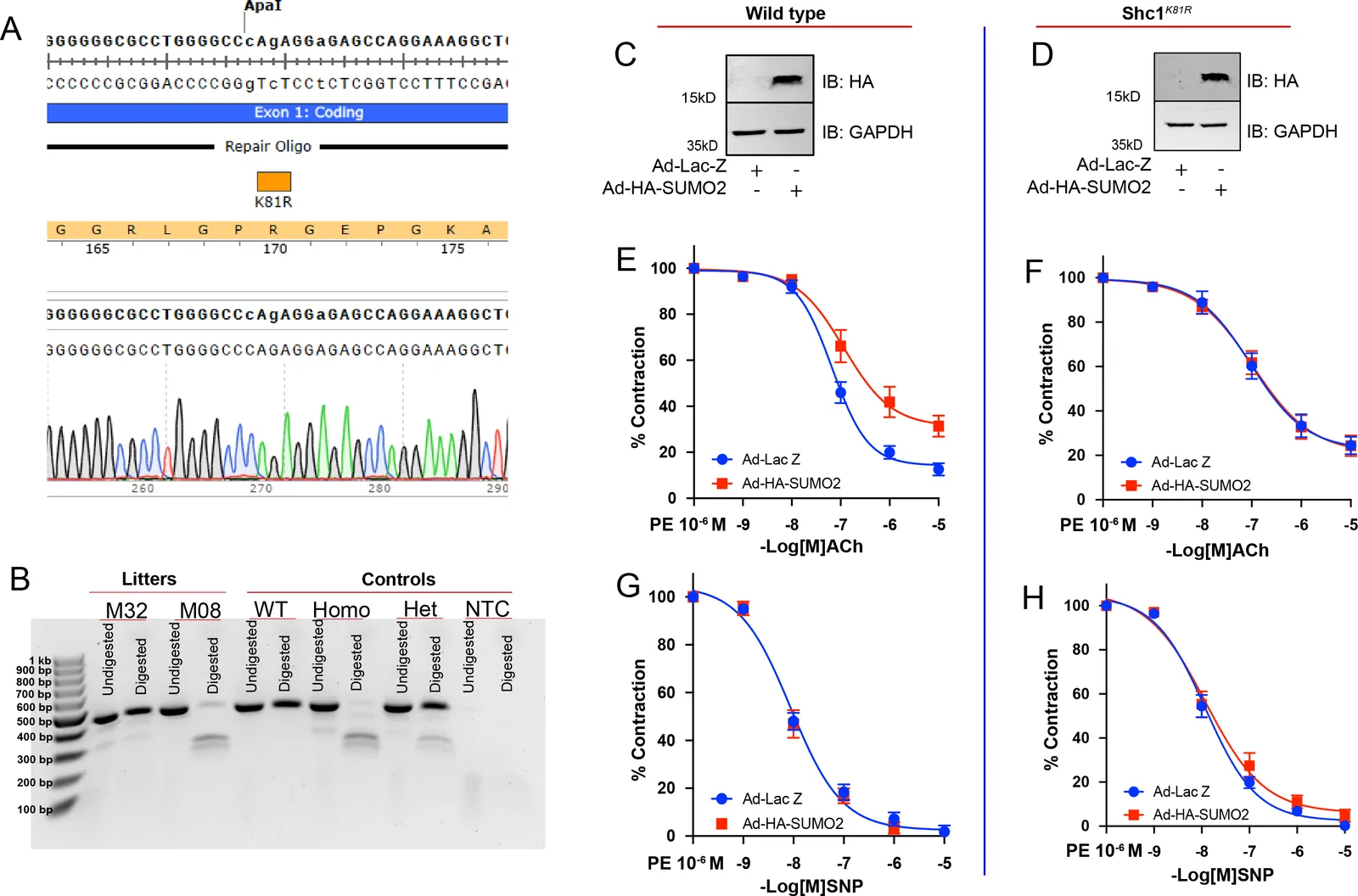
Shc1*^K81R^* are resistant to small ubiquitin-like modifier 2 (SUMO2)-induced endothelial dysfunction. (**A**) DNA sequencing data from a Shc1*^K81R^* mouse showing a point mutation (A>G) resulting in an amino acid change (K→R), and insertion of silent mutation to create a digestion site for ApaI endonuclease for genotyping. (**B**) DNA gel electrophoresis of a PCR-amplified and ApaI-digested amplicon identifying the Shc1*^K81R^* allele. (**C, D**) Representative immunoblots showing SUMO2 expression in aortic rings from wild-type and Shc1*^K81R^* mice (1.67×10^8^ pfu/ring). (**E, F**) Graphs showing % contraction following induction of endothelium-dependent relaxation in aortic rings from (**E**) wild-type mice, n=14–16 from 4 mice and (**F**) Shc1*^K81R^* mice (n=15–16 rings from 4 mice) expressing a control vector (Ad-Lac Z) or overexpressing SUMO2 (Ad-HA-SUMO2). (**G, H**) Graphs showing % contraction following induction of endothelium-independent relaxation in aortic rings from (**G**) wild-type (n=14–16 from 4 mice) and (**H**) Shc1*^K81R^* mice (n=14–15 from 4 mice) expressing a control vector (Ad-Lac Z) or overexpressing SUMO2 (Ad-HA-SUMO2). Curves were compared for nonlinear fit. ****p<0.0001. Ach, acetylcholine; SNP, sodium nitroprusside; PE, Phenylephrine; Homo, homozygous; Het, heterozygous; and NTC, No template control. Data represents mean ± SEM. Figure 6—source data 1.PDF file containing original western blots for Figure 6B. Figure 6—source data 2.Original files for western blot analysis displayed in Figure 6B. Figure 6—source data 3.PDF file containing original western blots for Figure 6C. Figure 6—source data 4.Original files for western blot analysis displayed in Figure 6C. Figure 6—source data 5.PDF file containing original western blots for Figure 6D. Figure 6—source data 6.Original files for western blot analysis displayed in Figure 6D.

To determine whether SUMO2ylation of p66Shc at lysine 81 modulates endothelial control of vascular tone, we generated a knock-in mouse model in which endogenous p66Shc was replaced with a SUMOylation-deficient mutant (p66ShcK81R). This model was generated using CRISPR-Cas9-mediated genome editing to substitute arginine for lysine at position 81 of p66Shc (Figure 6A and B). These mice are viable and, there was no obvious physiological difference from wild-type mice, except that the endothelium-independent relaxation of aortic rings was more pronounced in p66ShcK81R knock-in mice (Figure 6—figure supplement 1A–F). To examine the effect of SUMO2 on endothelial function in these mice, we used adenoviruses to express SUMO2 in aortic rings from WT and p66ShcK81R knock-in mice (Figure 6C and D). Subsequently, the aortic rings were evaluated for endothelium-dependent relaxation (induced by acetylcholine) and endothelium-independent relaxation (induced by sodium nitroprusside). In aortic rings from WT mice, endothelium-dependent relaxation was significantly delayed by SUMO2 overexpression (Figure 6E) whereas it was unchanged in p66ShcK81R mice (Figure 6F). In contrast, endothelium-independent relaxation was not affected by SUMO2 overexpression (Figure 6G and H). This suggests that the effect of SUMO2 on endothelial function is primarily mediated via p66ShcK81.

**Figure 6—figure supplement 1.**
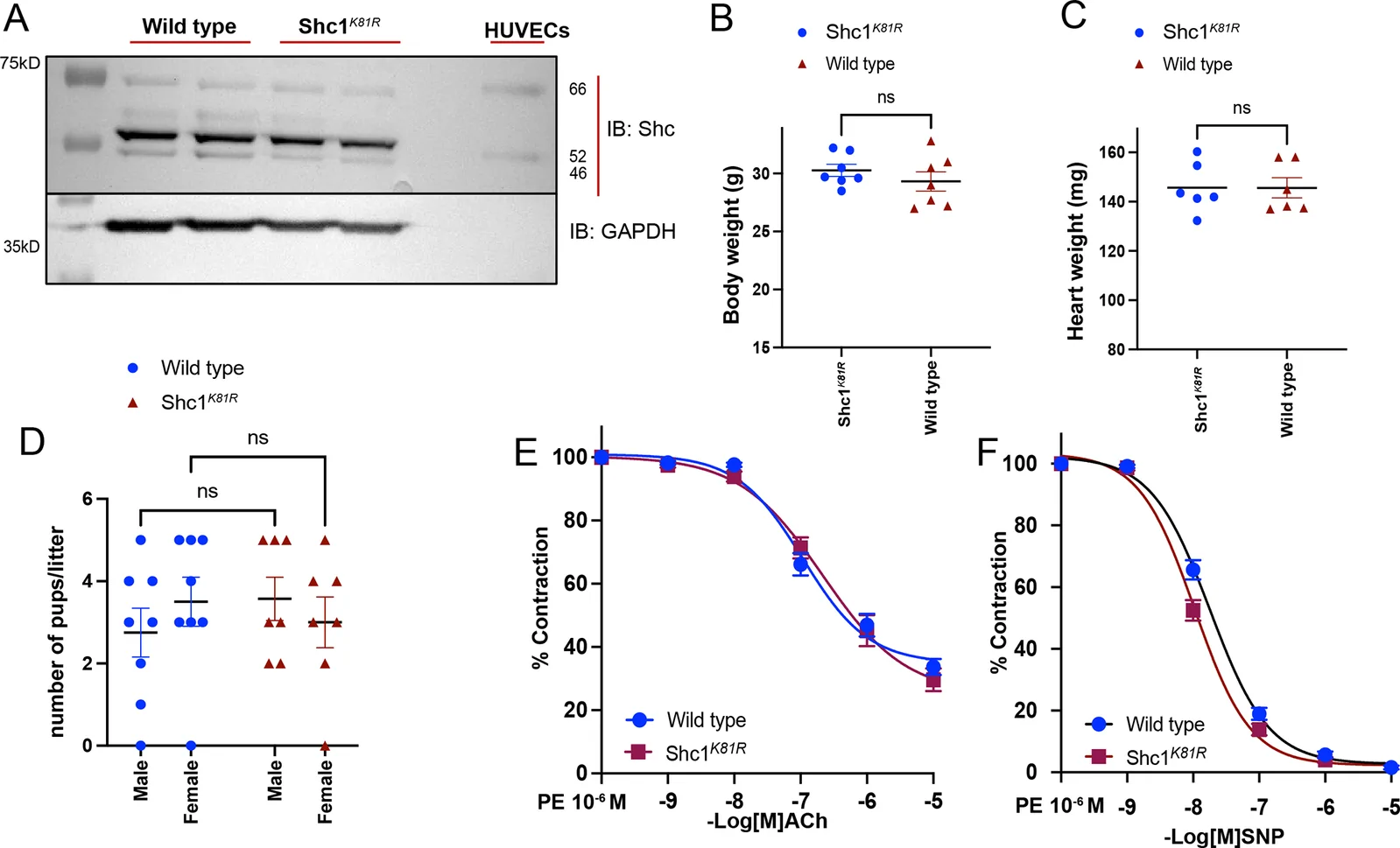
Physiological characterization of Shc1*^K81R^* mice. (**A**) Immunoblot for p66Shc isoforms in aortic lysates from wild-type and Shc1*^K81R^* mice. (**B**) Body weight of Shc1*^K81R^* and wild-type mice (n=7/group). (**C**) Heart weight of Shc1*^K81R^* and wild-type mice (n=6/group). (**D**) Litter-size of Shc1*^K81R^* (n=7) and wild-type mice (n=8). Graphs showing % contraction following induction of (**E**) endothelium-dependent and (**F**) endothelium-independent relaxation in aortic rings from wild-type mice, (n=15 rings from 4 mice) and Shc1*^K81R^* mice (n=15 rings from 6 mice). Curves were compared for nonlinear fit. ***p<0.001. ns = not significant. Ach, acetylcholine; SNP, sodium nitroprusside; PE, Phenylephrine. Data represents mean ± SEM. Figure 6—figure supplement 1—source data 1.PDF file containing original western blots for Figure 6—figure supplement 1A. Figure 6—figure supplement 1—source data 2.Original files for western blot analysis displayed in Figure 6—figure supplement 1A.

### P66ShcK81R knock-in mice are protected against hyperlipidemia-induced endothelial dysfunction

**Figure 7.**
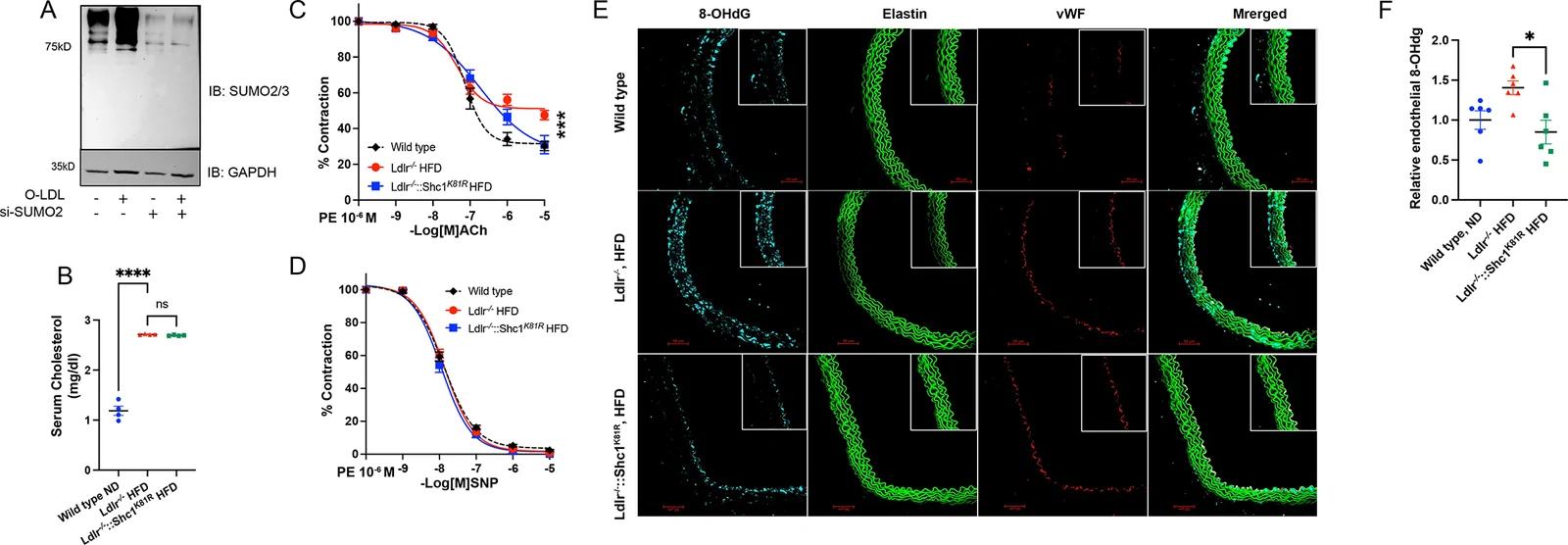
p66ShcK81R knock-in (KI) prevents hyperlipidemia-induced endothelial dysfunction and oxidative stress. (**A**) Immunoblot showing oxidized-LDL (o-LDL)-induced changes in small ubiquitin-like modifier 2/3 (SUMO2/3)-conjugated proteins in human umbilical vein endothelial cells (HUVECs) with and without SUMO2. (**B**) Graph showing serum cholesterol-level in wild-type, Ldlr*^-/-^* mice on a high fat diet, and Shc1*^K81R^* mice on a high fat diet (n=4/group). One-way ANOVA, ****p<0.0001. Graphs showing % contraction following induction of endothelium-dependent relaxation (**C**) and endothelium-independent relaxation (**D**) in aortic rings from male wild-type mice, (n=14 rings from 5 mice), Ldlr*^-/-^* mice on a high fat diet (n=18 rings from 6 mice) and Ldlr^-*/-*^:: Shc1*^K81R^* mice on a high fat diet (n=12 rings from 6 mice). Two-way ANOVA. ***p<0.001. (**E**) Representative images showing the level of 8-OHdG in aortic section of Ldlr*^-/-^* mice on a high fat diet (n=6 mice) and Ldlr*^-/-^*:: Shc1*^K81R^* mice on a high fat diet (n=6 mice) and their (**F**) quantification. One-way ANOVA. *p<0.05. HFD, high fat diet; Ach, acetylcholine; SNP, sodium nitroprusside; PE, Phenylephrine; 8-OHdG, 8 hydroxy deoxyguanosine. Data represents mean ± SEM. Figure 7—source data 1.Source data for immunoblots in Figure 7A. Figure 7—source data 2.Original datasets for Figure 7A.

To examine the physiological significance of p66Shc-SUMO2, we chose hyperlipidemia as a suitable stimulus, as it has been shown to promote sumoylation (Woo and Abe, 2010). To confirm hyperlipidemia-induced activation of sumoylation and involvement of SUMO2 specifically, we knocked down SUMO2 in HUVECs and treated them with oxidized low-density lipoprotein (ox-LDL). Immunoblotting with anti-SUMO2/3 antibody showed a robust increase in sumoylation with ox-LDL and the majority of sumoylation was due to SUMO2 (Figure 7A). Next, we crossbred Shc1*^K81R^* mice with low-density lipoprotein receptor knockout (LDLr^-/-^) mice to generate Ldlr*^-/-^*:: Shc1*^K81R^* mice and fed them a high-fat diet (HFD) for 4 weeks to induce hyperlipidemia. There was no difference in serum cholesterol levels between the Ldlr*^-/-^* and Ldlr*^-/-^*:: Shc1*^K81R^* mice on HFD (Figure 7B). We performed a vascular reactivity assay on the aortic rings of WT mice, Ldlr*^-/-^* mice on normal diet and Ldlr*^-/-^* on HFD and noted a significant impairment of endothelium-dependent relaxation of aortic rings from Ldlr*^-/-^* mice compared to aortic rings from wild-type mice and Ldlr*^-/-^*::Shc1*^K81R^* (Figure 7C). However, the endothelium-independent relaxations were similar among the groups (Figure 7D). Further, we evaluated the levels of oxidative stress in aortas of Ldlr*^-/-^* and Ldlr*^-/-^*::Shc1*^K81R^* mice on HFD by determining the level of 8-OHdG (8-hydroxy deoxyguanosine), a marker of oxidative damage to DNA. We noticed that Ldlr*^-/-^*::Shc1*^K81R^* mice have significantly less oxidative damage to aortic endothelium compared to Ldlr*^-/-^* mice (Figure 7E and F). Collectively, our study shows that p66Shc mediates the effects of SUMO2 on vasculature.

### p66ShcK81-SUMO2ylation affects multiple signaling pathways in endothelial cells

**Figure 8.**
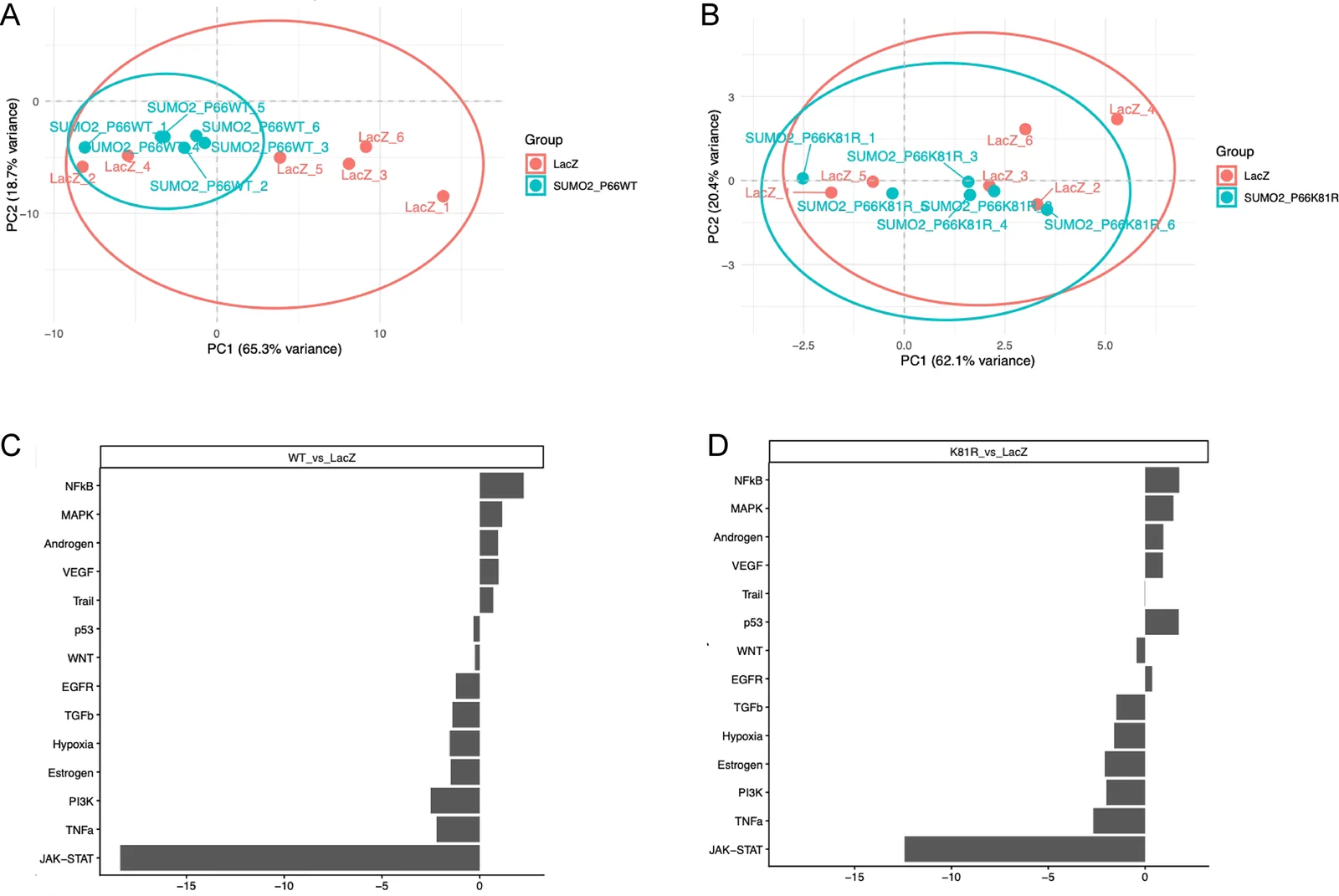
p66ShcK81 sumoylation affects multiple key regulatory pathways in endothelial cells. (**A, B**) Principal component analysis (PCA) of proteins expressed in endothelial cells expressing SUMO2+p66ShcWT and SUMO2+p66ShcK81R. (**C, D**) Bar chart presentation of key regulatory pathways differentially affected by small ubiquitin-like modifier 2 (SUMO2)-p66ShcWT (**C**) and SUMO2-p66ShcK81R (**D**) in endothelial cells. The protein expression data (fold change) were obtained from human umbilical vein endothelial cells (HUVECs) expressing SUMO2 with p66ShcWT or p66ShcK81R compared to those expressing Ad-Lac Z. Pathway enrichment analysis (**B**) was performed using R-based bioinformatics tools, including PROGENy/decoupleR pathway activity inference and visualization packages. Differentially regulated pathways associated with senescence, mitochondrial dysfunction, apoptosis signaling, and angiogenesis are shown. Data were derived from a sample size of n=6.

Given that SUMO2 modifies p66Shc at K81 and causes endothelial dysfunction, we next asked how p66ShcK81-SUMO2 affects endothelial cells. We performed global proteomics profiling of HUVECs overexpressing SUMO2 and p66ShcWT or p66ShcK81R using mass spectrometry. Comparative analysis of protein expression was performed against control (HUVECs infected with an equal number of adenoviruses expressing LacZ) “Figure 8—figure supplement 1A”. Principal component analysis showed a distinct protein expression profile for cells expressing p66ShcWT and p66ShcK81R (Figure 8A and B). To understand the effect of p66Shc K81 SUMOylation, we examined pathways altered in cells expressing p66ShcWT and p66ShcK81R. The majority of pathways were downregulated, with particularly strong suppression of JAK-STAT signaling. This observation is consistent with the known role of Shc proteins in mediating growth factor receptor signaling. Unlike p52Shc and p46Shc, p66Shc is reported to negatively regulate growth factor receptor signaling. Supporting this, our mass spectrometry data revealed a greater than 15-fold downregulation of JAK-STAT signaling in cells expressing p66ShcWT in the presence of SUMO2 (Figure 8C and D). In contrast, this effect was attenuated in cells expressing p66ShcK81R with SUMO2 (Figure 8C and D, Figure 8—figure supplement 1B). It is important to note that endogenous p66ShcWT was present in both conditions, which may have competed with the overexpressed p66ShcK81R. Together, these findings suggest that K81 SUMOylation plays a critical role in regulating p66Shc function.

**Figure 8—figure supplement 1.**
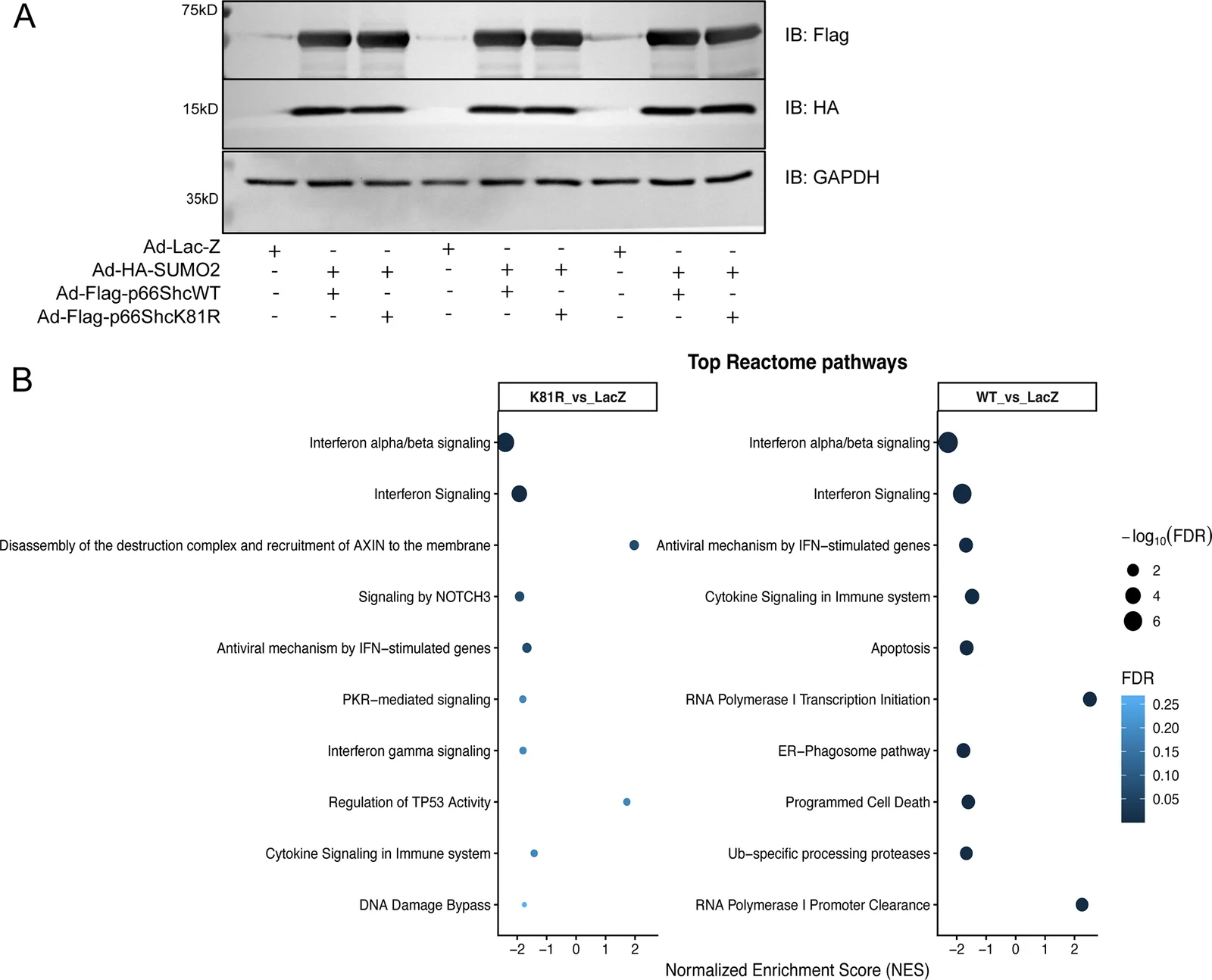
P66Shck81-SUMO2ylation induced changes in signaling pathways in human umbilical vein endothelial cells (HUVECs). (**A**) Representative immunoblots of endothelial cell lysate expressing Flag-p66ShcWT, Flag-p66ShcK81R, and HA-SUMO2 (n=3 shown, n=6 performed). (**B**) Bubble chart representation of all the regulatory pathways impacted by SUMO2-p66Shc in endothelial cells, rescued by p66ShcK81R. Pathway enrichment analysis was performed using R-based Reactome pathway analysis and visualization packages on differential protein expression data obtained from HUVECs expressing SUMO2-p66ShcWT or SUMO2-p66ShcK81R compared with Ad-LacZ controls (n=6). Figure 8—figure supplement 1—source data 1.PDF file containing original western blots for Figure 8—figure supplement 1A. Figure 8—figure supplement 1—source data 2.Original files for western blot analysis displayed in Figure 8—figure supplement 1A.

## Discussion

Although SUMO2 and p66Shc are expressed in endothelium and are associated with ED, the mechanisms by which this occurs are unclear. Here, we identified SUMO2 modification of p66Shc as a novel molecular signal regulating vascular endothelial function. SUMO2 modifies p66Shc at K81, which increases phosphorylation of p66Shc at S36, resulting in its translocation to the mitochondria. These steps are critical for p66Shc-dependent ROS production. Notably, this phenomenon was evident in non-endothelial cells as well, suggesting that SUMO2-p66Shc may also regulate other physiological functions. Mice expressing a non-sumoylatable form of p66Shc (Shc1*^K81R^*) were protected against SUMO2- and hyperlipidemia-induced ED and oxidative stress, which further supports the role of SUMO2-p66ShcK81 in regulating endothelial function.

Multiple studies suggest that sumoylation promotes ED (Abe et al., 2017). Preventing sumoylation by downregulating the SUMO ligases Ubc9 (E2 ligase), and PIAS1 (E3 ligase) rescued oxidative stress and advanced glycation end product-mediated endothelial inflammation and dysfunction (Woo et al., 2008). Conversely, partial genetic deletion of the de-sumoylation enzyme sentrin/SUMO-specific protease 2 (SENP2), significantly increased endothelial apoptosis, inflammation, and dysfunction, which promoted atherosclerotic plaque formation (Heo et al., 2015; Heo et al., 2013). However, the isoform-specific effects of SUMOs on endothelial function have not been well studied. We previously reported that endothelial expression of SUMO2 impairs vasorelaxation (Kim et al., 2019). By showing that mice having p66ShcK81R knock-in are resistant to endothelial effects of SUMO2 and hyperlipidemia, this study explains the prior findings that the effects of SUMO2 on vasculature are likely mediated by p66Shc.

The adapter protein p66Shc mediates receptor tyrosine kinase signaling in a variety of cell types. Among them, the effects of p66Shc on endothelial cells are well studied and pronounced (Kumar, 2019). In particular, endothelial function in mice with genetic deletion of p66Shc is protected against a variety of stimuli, such as diabetes, aging, and hyperlipidemia (Napoli et al., 2003; Camici et al., 2007; Francia et al., 2004). p66Shc produces ROS by oxidation of cytochrome c in the mitochondria as well as by activating Rac1 (Khanday et al., 2006; Giorgio et al., 2005). Phosphorylation at S36 in the CH2 domain of p66Shc is one of the earliest discovered posttranslational modifications that governs its oxidative function (Pinton et al., 2007). Crosstalk among different posttranslational modifications, including phosphorylation, acetylation, and sumoylation, is well known (Chang and Abe, 2016; Abe and Berk, 2014; Stankovic-Valentin et al., 2007; Wu and Chiang, 2009). Our finding that SUMO2 increases S36 phosphorylation in p66Shc suggests the existence of such crosstalk in regulating the oxidative function of p66Shc. The molecular weight of the phosphorylated p66ShcS36 protein band (close to 70 kDa) suggests that the pool of SUMO2-induced phosphorylated p66ShcS36 is not SUMO2ylated. Therefore, it is likely that K81-SUMOylated and S36-phosphorylated forms of p66Shc coexist in the same pool of protein, but that p66Shc is not SUMOylated and phosphorylated at the same time. Future studies will be needed to establish the hierarchy of this phenomenon.

Based on mass spectrometry analysis and in vitro sumoylation of recombinant p66Shc, it appears that p66ShcK81 is a primary target of SUMO2. However, the multiple protein bands higher than 66 kDa that were present in the immunoprecipitation assay suggest the possibility that additional sumoylation sites are present in p66Shc. Technical challenges limited our ability to determine the nature of these protein bands. Yet, in silico analysis using an available sumoylation prediction tool (Cuckoo workgroup) indicates three possible sumoylation motifs present in p66Shc, namely K81, K462, and K582, which can be non-specifically modified by SUMO1/2/3/4. Importantly, among the possible sumoylation motifs only K81 is present in the CH2 domain, which is a unique structural feature of p66Shc (i.e. not present in other isoforms: p52Shc and p46Shc) that has a regulatory effect on its function. Therefore, even though other lysines are sumoylated, it is likely that the effect of p66Shc sumoylation is mediated through the K81 modification and has also been supported by proteomics analysis.

The primary function of the ShcA family of adaptor proteins is to mediate growth factor receptor signaling (Kumar, 2019). However, p66Shc is unique within this family, as it deviates from the classical role of Shc proteins and instead promotes oxidative stress. Our global mass spectrometry data suggest that enhancing p66ShcK81 SUMO2ylation inhibits JAK-STAT signaling, a key downstream pathway of growth factor receptor signaling. The negative regulation of growth factor receptor signaling by p66Shc, through competition with p52Shc for Grb2 binding, has been previously reported (Xi et al., 2008). In addition, a recent study demonstrated that p66Shc can mediate EGFR-ERK signaling (Powell et al., 2025). Therefore, beyond its established role in ROS regulation, p66ShcK81-SUMO2ylation may have additional functional roles in endothelial cell physiology.

One of the limitations of this study is that we did not perform gain-of-function experiments to ascertain if SUMO2-modified p66ShcK81 regulates p66Shc function in vivo, which is technically too challenging. Additionally, we were unable to examine the behavior of the phosphomimetic double mutant (S36D/K81R) to directly assess the interplay between K81 SUMO2ylation and S36 phosphorylation in mitochondrial localization. Prior studies have shown that K81 of p66Shc undergoes acetylation (Kumar et al., 2017) and that a specific lysine can undergo sumoylation as well as acetylation (Stankovic-Valentin et al., 2007). Although p66ShcK81R disrupts acetylation and sumoylation that occur from acetyltransferase and SUMOylating enzymes, it is possible that the same lysine is sumoylated and acetylated in a context-dependent manner. Importantly, the extent of posttranslational modification is so small that competition between acetylation and sumoylation for the same target residue is highly unlikely. The fact that Shc1*^K81R^* mice are protected against SUMO2-induced ED supports the hypothesis that p66ShcK81 mediates the endothelial effects of SUMO2.

Collectively, our study identified p66Shc SUMO2ylation as a unique cellular signaling mechanism specifically regulating vascular endothelial function. As such, this signaling mechanism may also affect advanced pathologies, such as atherosclerosis, where both increased SUMOylation and expression of p66Shc have been reported. Nevertheless, given its potential to regulate endothelial function, SUMO2ylation of p66Shc presents a novel therapeutic target for pathologies associated with ED.

## Materials and methods

**Key resources table:** 

Reagent type (species) or resource	Designation	Source or reference	Identifiers	Additional information
Genetic reagent (*Mus musculus*)	Shc1^K81R^ mice	This paper	Shc1^K81R^	Whole-body knock-in of Shc1^K81R^
Genetic reagent (*M. musculus*)	LDLr^-/-^ mice	Jax	B6.129S7-Ldlrtm1Her/J	LDL receptor knockout mice
Cell line (*Homo sapiens*)	HEK-293T	ATCC	Cat# CRL11268	Shared by Dr. Dale E. Abel
Cell line (*H. sapiens*)	HUVEC	promocell	Cat# C1220	Ordered through Fisher Scientific
Cell line (*H. sapiens*)	HUVEC	Lonza	Catalog #: C2519A	HUVEC - Human Umbilical Vein Endothelial Cells, Pooled
Antibody	anti-SUMO2/3 (Rabbit polyclonal)	Sigma-Millipore	Cat# 633306	WB (1:1000)
Antibody	anti-SUMO2/3 (Rabbit polyclonal)	CST	Cat# 4971	WB (1:1000)
Antibody	Anti-GAPDH	CST	Cat# 2118 S	WB (1:1000)
Antibody	Anti-HA (Mouse monoclonal)	CST	Cat# 3724 s	WB (1:1000)
Antibody	Anti-His (Mouse monoclonal)	CST	Cat# 2366 S	WB (1:1000)
Antibody	Anti-Ubc9 (Rabbit Monoclonal Antibody)	CST	Cat# XP 4786 s	WB (1:1000)
Antibody	Anti-Shc	BD	Cat # 610879	WB (1:2000)
Antibody	anti-human phopsho-p66ShcS36 (Mouse monoclonal)	Abcam	Cat# Ab267413	WB (1:1000) (This product is discontinued from abcam)
Antibody	anti-human SUMO2-p66ShcK81 (Rabbit polyclonal)	This paper	YenZym, CA	Detects p66Shc-sumoylation on non K81 motif as well
Antibody	anti-Flag (Mouse-monoclonal)	Sigma-Millipore	Cat# F1804	WB (1:1000)
Antibody	anti-COXIV (Rabbit monoclonal)	CST	Cat# 4850 S	WB (1:1000)
Recombinant DNA reagent	HA-SUMO2 (plasmid)	This paper		Kumar Lab
Recombinant DNA reagent	Flag-p66Shc (plasmid)	This paper		Kumar Lab
Recombinant DNA reagent	FNpCDNA3	Dr. Robert Oshima	Addgene plasmid # 45346	
Recombinant DNA reagent	Flag-p66ShcK81R	This paper		Kumar Lab
Recombinant DNA reagent	Flag-SENP1	Dr. Edward Yeh	Addgene plasmid # 17357	
Recombinant DNA reagent	Ubc9	Dr. Edward Yeh	Addgene plasmid # 20082	
Recombinant DNA reagent	Ad-Flag-p66ShcWT	This paper		Kumar Lab
Recombinant DNA reagent	Ad-Flag-p66ShcK81R	This paper		Kumar Lab
Recombinant DNA reagent	Ad-HA-SUMO2	This paper		Kumar Lab
Recombinant protein	Recombinant His-p66ShcWT	This paper		Kumar Lab
Recombinant protein	Recombinant His-p66ShcK81R	This paper		Kumar Lab
Commercial assay or kit	SUMO conjugation kit	Enzo	BML-UW8955-0001	
Commercial assay or kit	Total cholesterol assay kit	Cell Biolab	STA-390	
Other	Special diet a high fat diet	Envigo	TD.88317	High fat diet
Other	Genotyping p66ShcK81R KI mice genotyping primer Forwards	This paper	PCR primers	CTT CGG AAT GAG TCT CTG TCA TC
Other	Genotyping p66ShcK81R KI mice genotyping primer Forwards	This paper	PCR primers	ACC CGA ACC AAG TAG GAA ACG
Sequence-based reagent	Shc1	Thermo Fisher	Catalog #: 4390815 siRNA ID: s73683	Silencer Select Pre-Designed siRNA
Sequence-based reagent	SUMO2	Thermo Fisher	Catalog #: 4392421 siRNA ID: s13180	Silencer Select Pre-Designed siRNA
Sequence-based reagent	siRNA: nontargeting control	Thermo Fisher	4390844	Silencer Select
Other	MitoSOX	Thermo Fisher	M36008	Mitochondrial Superoxide Indicator. Final working concentration: 2 µM
Other	H_2_DCFDA	Thermo Fisher	D399	Indicator for reactive oxygen species Final working concentration: 25 µM

### Animals

All experimental animal procedures were approved by the Institutional Animal Care and Use Committee at the University of Iowa and conformed to the Guide for the Care and Use of Laboratory Animals published by the National Institutes of Health. Wild-type mice (C57BL/6) were procured from Jackson Laboratory. The p66ShcK81R knock-in mice were generated with the help of the Genome Editing Core Facility, University of Iowa, Iowa City. Using CRISPR-Cas9 technology, a mutation was introduced in embryos from C57BL/6 J mice and the embryos were implanted into pseudopregnant females. Founders were identified by PCR genotyping and Sanger sequencing. Subsequently, mice were inbred to generate homozygous p66ShcK81R knock-in mice.

To generate Ldlr*^-/-^*:: Shc1*^K81R^* mice, Ldlr*^-/-^* mice (B6.129S7-*Ldlr^tm1Her^*/J) were obtained from Jackson Laboratory and crossbred to Shc1*^K81R^* mice to generate Ldlr*^-/-^*:: Shc1*^K81R^* mice homozygous for both Ldlr deletion and Shc1*^K81R^* knock-in. To induce hyperlipidemia, these mice were fed on a high fat diet (Envigo-TD.88317) for 4 weeks. Serum cholesterol was measured using a commercially available kit (Cell Biolab, #STA-390).

### Genotyping protocol for p66ShcK81R

DNA was isolated from ear punches of mice using a commercially available DNA isolation kit (NucleoSpin Tissue DNA, Macherey-Nagel GmbH & Co.). Polymerase chain amplification (PCR) was performed to amplify the DNA sequence around the knock-in position using a Phusion high-fidelity DNA polymerase master mix reaction (NEB); forward primer (5**′**- CTT CGG AAT GAG TCT CTG TCA TC-3**′**) and reverse primer (5**′**- ACC CGA ACC AAG TAG GAA ACG-3**′**). The reaction mixture was preheated at 70 °C for 2 min followed by initial denaturation at 98 °C for 2 min, then 35 cycles at 98 °C for 20 s, 62 °C for 30 s, 72 °C for 40 s, and a final extension at 72 °C for 5 min. PCR amplified products were digested with ApaI enzyme at 37 °C for 2.5 hr. Agarose gel electrophoresis was performed to determine the amplification and digestion pattern of the amplicon. Mice identified by genome sequencing were used as a positive control.

### Cell culture

Human umbilical vein endothelial cells (HUVECs) were purchased from PromoCell (Heidelberg, Germany) and Lonza (Walkersville, MD) and were cultured in endothelial growth medium-2 from PromoCell and Lonza. HEK293 cells were cultured in DMEM (Gibco) supplemented with 10% fetal bovine serum (Thermo) and 1% Pen Strep (penicillin 10,000 units/ml and 10,000 ug/ml streptomycin; Gibco).

### Reagents

Primary antibodies: Monoclonal Flag (Catalog no. F1804, Sigma, MO); Monoclonal GAPDH (Catalog no. 2118 S, Cell Signaling Technology, MA); monoclonal Senp1 (Catalog no. sc-271360, Santa Cruz Biotechnology, TX); Monoclonal HA (Catalog no. 3724 s Cell Signaling Technology); Monoclonal His-tag (Catalog no. 2366 S Cell Signaling Technology); Polyclonal Phospho-p66Shc (Catalog no. Ab267413 Abcam, MA); SUMO2/3 (Catalog no. 633306, Sigma and Catalog no. 4971, Cell Signaling Technologies); Monoclonal Ubc9 (D26F2) (Catalog no. XP 4786 s, Cell Signaling Technology); PhosphoDetect Anti-Shc/p66 (Catalog no. 566807, MilliporeSigma, MO); Cox IV (3E11) (Catalog no. 4850 S, Cell Signaling Technology); sumoylation kit (BML-UW8955-0001, Enzo Life Sciences, NY), Total cholesterol assay kit (Catalog no. STA-390, Cell Biolabs).

Secondary antibodies: polyclonal IR-dye 800 Rb (Catalog no. ab216773, Abcam); polyclonal IRdye 680 (Catalog no. ab216776, Abcam); anti-rabbit IgG HRP-linked (Catalog no. 7074 S, Cell Signaling Technology); anti-mouse IgG HRP-linked (Catalog no. 7076 S, Cell Signaling Technology).

### Plasmids

We generated a new construct of p66Shc (human) with a Flag tag (DYKDDDDK) at the N-terminus (Flag-p66Shc) in the pcDNA3 backbone. FNpcDNA3 was a gift from Dr. Robert Oshima (Addgene plasmid # 45346). We also generated HA-tagged (MYPYDVPDYA) SUMO2 (Human) in the same backbone. Successful cloning was verified by DNA sequencing. FLAG-SENP1 (Addgene plasmid # 17357) Cheng et al., 2007 and Ubc9 (Addgene plasmid # 20082) Gong et al., 1997 were gifts from Dr. Edward Yeh.

### Adenoviruses

Adenoviruses of Flag-p66ShcWT, Flag-p66ShcK81R, and HA-SUMO2 were generated with the help of the Viral Vector Core Facility, University of Iowa. Target sequences were cloned into a shuttle vector. For adenoviral expression, Flag-p66ShcWT, Flag-p66ShcK81R, and HA-SUMO2 were cloned into a shuttle vector plasmid (G0688pacAd5CMV) provided by the Viral Vector Core facility. Constructs were verified for proper inserts by digestion followed by Sanger sequencing. Further processing was supported by the Viral Vector Core facility.

### Mutagenesis of p66Shc

To create p66ShcK81R, we performed site-directed mutagenesis on Flag-p66Shc to induce an AAG-to-AGA mutation. The following primers were used: forward 5**′**-cctggctcccctctagaccctgggcgcc-3**′** and reverse 5**′**-ggcgcccagggtctagaggggagccagg-3**′**. Successful mutagenesis was verified by DNA sequencing.

### Adenoviral expression

An optimal volume of viral stock was added to the cells with growth medium. We used an MOI of 1:100 in most of the experiments. The Specific MOI is described in the figures. Cells were analyzed after approximately 40 hr of infection.

### Plasmid and siRNA transfection

For plasmid transfection, Lipofectamine 2000 (Invitrogen, MA) was used as per the manufacturer’s instructions. For 1 μg of plasmid, we used 2 μl Lipofectamine. p66Shc-specific siRNAs (custom-made) and a non-specific control siRNA were purchased from Invitrogen. An appropriate amount of siRNA was incubated with Lipofectamine 2000 (Invitrogen) at room temperature for 20 min before being added to cells prepared with Opti-MEM medium (Invitrogen). Four hours later, the medium was replaced with fresh endothelial growth media and cells were cultured for an additional 48 hr.

### Recombinant protein expression and purification

Purified recombinant protein was synthesized as published earlier with slight modification (Yadav et al., 2017). DNA sequences coding His-p66ShcWT and His-p66ShcK81R proteins were PCR-amplified from Flag-p66ShcWT and Flag-p66ShcK81R plasmids. PCR-amplified His-p66ShcWT and His-p66ShcK81R were cloned into the pET-21a expression vector. Sanger sequencing confirmed the presence of the insert. Sequenced plasmids were transformed into Rosetta 2 (DE3) cells and inoculated into 50 ml of LB broth supplemented with 50 μl ampicillin (100 μg/ml) and 50 μl chloramphenicol (34 μg/ml) antibiotics. The cells were then incubated overnight at 37 °C at 250 rpm for growth. For secondary inoculum, 10 ml of the primary culture was inoculated into 1 l of 2xYT media (Yeast-Tryptone) and incubated at 37 °C until the O.D. reached 0.6 at 600 nm. Cultures were then cooled at 2–8°C for a minimum of 20 min, followed by 5 min at room temperature. Induction of the culture was done using 0.250 mM IPTG, and the incubation continued at 16 °C, 200 rpm, overnight. The culture was pelleted by centrifugation at 4 °C. The cell pellets were sonicated on ice (five 30 s pulses). The lysed sample was centrifuged at 18,000 rpm for 1 hr at 4 °C. The supernatant was collected and passed through a pre-packed, pre-equilibrated Ni-NTA resin column for protein purification. Western blotting confirmed the presence of the target protein.

### Mass spectrometry for sumoylation and data analysis

Recombinant p66Shc was sumoylated with a commercially available sumoylation kit (Enzo Life Sciences). Sumoylated protein was resolved by SDS-PAGE. Gels were stained with mass spectrometry compatible silver stain (SilverQuest, Invitrogen). Gel pieces were excised based on the observed SUMOylation signal in the immunoblot of recombinant p66Shc. Gel pieces were destained and reduced with 5 μl of 200 mM tris(2-carboxyethyl)phosphine (TCEP) (1 hr incubation, 55 °C) and alkylated with 5 μl of 375 mM iodoacetamide (IAA) (30 min incubation in the dark, room temperature). Protein digestion was carried out using 2.5 μg of trypsin per sample (16 hr incubation, 37 °C). The next day, samples were dried using a speed vacuum and then desalted with C18 spin columns (Pierce, MA). Clean peptides were dried again with a speed vacuum and resuspended in 0.1% formic acid, and then next analyzed using a high-resolution mass spectrometry nano-LC-MS/MS Tribrid system, Orbitrap Fusion Lumos coupled with an UltiMate 3000 HPLC system (Thermo Fisher Scientific). Peptide identification was performed by searching MS/MS data against the p66Shc protein sequence in PEAKS Studio. We detected a missed cleavage at a lysine (K) residue, as shown in Figure 3B, which was followed by a QTGG modification with a mass of 343.15 Da. This specific mass shift was consistent with the expected tryptic peptide from sumoylated peptide, as shown by other studies (Wang et al., 2019; Chen et al., 2024).

### Label-free quantitative mass spectrometry and data analysis

HUVECs were infected with Ad-HA-SUMO2 and Ad-Flag-p66ShcWT or Ad-Flag-p66ShcK81R. Samples were lysed in RIPA buffer and protein expression was determined by immunoblotting. Whole-cell lysate containing 50 µg of protein per sample from six biological replicates per group was taken and detergent was removed by chloroform/methanol extraction, and the protein pellet was resuspended in 100 mM ammonium bicarbonate and digested with MS-grade trypsin (Pierce) overnight at 37 °C following reduction with 10 mM DTT at 56 °C for 30 min and alkylation using 50 mM iodoacetamide at RT for 25 min. Peptides were cleaned with PepClean C18 spin columns (Thermo) and were re-suspended in 2% acetonitrile (ACN) and 0.1% formic acid (FA) and then 500 ng of each sample was loaded onto trap column an Acclaim PepMap 100 75 µm × 2 cm C18 LC Columns (Thermo Scientific) at flow rate of 4 µl/min then separated with a Thermo RSLC Ultimate 3000 (Thermo Scientific) on a Thermo Easy-Spray PepMap RSLC C18 75 µm × 50 cm C18 2 µm column (Thermo Scientific) with a step gradient of 4–25% solvent B (0.1% FA in 80% ACN) from 10 to 100 min and 25–45% solvent B for 100–130 min at 300 nl/min and 56 °C with a 155 min total run time. Eluted peptides were analyzed by a Thermo Orbitrap Exploris 480 (Thermo Scientific) mass spectrometer in a data-dependent acquisition mode. A survey full scan MS (from m/z 350–1200) was acquired in the Orbitrap with a resolution of 60,000. The Normalized AGC target for MS1 was set as 300% and ion filling time set as 25 ms. The most intense ions with charge states 2–6 were isolated in 3 s cycle and fragmented using HCD fragmentation with 30% normalized collision energy and detected at a mass resolution of 15,000 at 200 m/z. The AGC target for MS/MS was set as 50% and ion filling time set to auto for 30 s with a 10 ppm mass window. Protein identification was performed by searching MS/MS data against the Swiss-Prot human protein database using the in-house PEAKS X+DB search engine. The search was set up for full tryptic peptides with a maximum of two missed cleavage sites. The precursor mass tolerance threshold was set 10 ppm for and maximum fragment mass error was 0.02 Da. The significance threshold of the ion score was calculated based on a false discovery rate of ≤1%. Quantitative data analysis was performed using Progenesis QI Proteomics 4.2 (Nonlinear Dynamics). Statistical analysis was performed using ANOVA and the Benjamini–Hochberg (BH) method was used to adjust p values for multiple-testing caused false discovery rate. The adjusted p≤0.05 was considered significant.

Principal Component Analysis (PCA) was employed to assess the global molecular differences induced by SUMO2_P66WT and SUMO2_P66K81R expression relative to the LacZ control. In both comparisons, PCA revealed distinct clustering of experimental groups, indicating substantial divergence in molecular profiles. For the SUMO2_P66WT group, PC1 accounted for 65.3% of the total variance, while PC2 explained 18.7%, together capturing 84% of the dataset’s variability. Similarly, in the SUMO2_P66K81R comparison, PC1 and PC2 explained 62.1 and 20.4% of the variance, respectively, totaling 82.5%. In both cases, biological replicates clustered tightly within their respective groups, underscoring the reproducibility of the data. These results demonstrate that both SUMO2_P66WT and SUMO2_P66K81R expression lead to pronounced and consistent alterations in the molecular landscape compared to LacZ, with each variant contributing distinct signatures of change.

### Whole-cell ROS assay by spectrophotometry

HUVECs were plated in clear-bottom black-walled 96-well plates and infected with adenoviruses encoding HA-SUMO2 or control virus encoding LacZ. For ROS detection and quantification, cells were incubated with either MitoSOX (2 µM, Life Technologies) or H_2_DCFDA (25 µM, Life Technologies) in EBM-2 for 30 min at 37 °C in CO_2_ incubator. Following incubation, cells were washed with pre-warmed EBM2 medium and maintained at 37 °C in a CO_2_ incubator for another 30 min. MitoSOX fluorescence was measured at 396/610 nm while DCF fluorescence was measured at 485/525 nm using a plate reader (ID3, Molecular Devices). Each well was measured at four different points at the 1 mm distance and the mean was considered as final reading for that well. Values were normalized against the background fluorescence. We excluded the first and last rows in each plate.

### Immunoprecipitation

Protein lysate was pre-cleared using Sepharose beads for 2 hr at 4 °C on the rocker. The supernatant was collected and incubated with the specified antibody (1 μg of antibody/mg protein) and Sepharose beads overnight at 4 °C. Sepharose beads were washed 8 times with ice-cold lysis buffer (1 ml each wash). After that, the beads were boiled for 5 min at 95 °C and subjected to SDS-PAGE.

### Generation of SUMO2-p66Shc antibody

The antibody to detect SUMO2-p66Shc was generated by using the branched peptide, mimicking the region of SUMO2 attachment (SUMO2-K81) to p66Shc as an immunogen using commercial source (YenZym, CA). The antibody was purified and tested for its specificity by ELISA and immunoblotting.

### Immunoblotting

Cells or tissues were washed with PBS and lysed in an appropriate amount of protein lysis buffer (Triton X-100: 1%, Tris pH 7.4: 50 mM, EDTA: 5 mM, NaCl: 150 mM, and glycerol: 5%) containing Protease Inhibitor Cocktail (Roche). We used Phosphatase Inhibitor Cocktail (Millipore) and the de-sumoylation inhibitor N-ethylmaleimide (20 mM) in the lysis buffer where appropriate. Cell lysis was performed by repeated freeze-thaw cycles followed by sonication for 25 s (5 s on, 2 s off, and 20% amplitude). The homogenate was centrifuged at 13,000 g for 20 min at 4 °C, and the supernatant was used for protein quantification.

We used approximately 30 μg of protein lysate. Cell lysate was mixed with protein loading dye and boiled at 95 °C for 5 min. Following brief centrifugation, lysates were loaded onto an acrylamide gel for SDS-PAGE. Protein was transferred to a nitrocellulose membrane. Membranes were treated with 5% milk or BSA in Tris-buffered saline with 0.1% Tween-20 and were probed with the specified primary antibody and the appropriate peroxidase-conjugated (Abcam) or fluorescent antibody (Abcam and Licor, NE) secondary antibody. Chemiluminescent signal was developed using SuperSignal West Femto substrate (Pierce, Rockford, IL). Images were acquired using a signal detection system (iBright 1500FL, Thermo) and quantified with iBright image analysis software.

### Mitochondrial Translocation

HUVECs were used to determine the mitochondrial localization of p66Shc. Cells were washed with cold 1× PBS buffer and collected in 500 μl MSE buffer (5  mM MOPS, 70  mM sucrose, 2  mM EGTA, 220  mM mannitol, pH 7.2). Maintaining the cell suspension at 4 °C, cells were homogenized using a smooth PTFE pestle in a glass tube until 85 to 95% of the cells were ruptured. Unbroken cells and nuclei were removed by centrifugation at 1000 g for 5 min. Supernatant was collected and centrifuged at 5000 g for 10 min to obtain the mitochondrial and cytosolic fractions. The mitochondrial pellet was further processed for protein lysate, as described under Immunoblotting.

### Ex vivo adenoviral infections and vascular reactivity

Ad-LacZ or Ad-HA-SUMO2 adenoviral stocks at a final concentration of 2.5×10^7^ pfu/ml in EGM-2 media were added to each aortic ring and incubated at 37 °C for 24 hr. Infected vessels were suspended between two wire stirrups (150 mm) in a four-chamber myograph system (DMT Instruments) in 6 ml Krebs-Ringer (95% O_2_: 5% CO_2_, pH 7.4, 37 °C). All concentration-effect curves were performed on arterial rings beginning at their optimum resting tone. Endothelium-dependent and independent relaxation was determined by generating dose-response curves to acetylcholine (ACh 10^–9^–10^–5^ M) and sodium nitroprusside (SNP 10^–9^–10^–5^ M), respectively, on phenylephrine (PE 10^–6^ M)-induced precontracted vessels. Vasorelaxation evoked by ACh and SNP was expressed as percent contraction with reference to phenylephrine (PE 10^–6^ M) and determined by calculating the percentage of inhibition to the pre-constricted tension.

### Histological processing and quantification

For immunohistochemical examinations, 10 μm thick sections were prepared from cryopreserved aortic rings. Sections were washed with PBS (three times) and were blocked with normal goat serum (Millipore) for 30 min at room temperature. Next, the blocking medium was removed, primary antibody was added and sections were incubated at 4 °C. After incubation with primary antibodies, the sections were incubated with secondary antibodies tagged with fluorescent dye. Images were acquired on a confocal microscope (Zeiss 710). Images were quantified using ZEN Blue software (Zeiss). For endothelial-specific measurements, the endothelial layer was manually traced following the lead of the innermost elastic lamina and staining for endothelial-specific von Willebrand factor. Multiple images were captured from each section and the mean fluorescence intensity was calculated for each image which was averaged for each mouse, and expressed as fold differences among groups. Autofluorescence of elastin lamina was captured to clearly define the endothelial-specific fluorescence.

### Statistics

All data met the assumptions of the statistical tests, and all statistical analyses were performed using GraphPad Prism 9 unless specified. Data represent the mean ± SEM of at least three independent assays. Normal distribution was checked using the Kolmogorov–Smirnov test. Significance of differences between two groups was determined using a two-tailed independent-sample Student’s *t*-test. One-way ANOVA was used for comparing more than two groups and two-way ANOVA was used for comparing two variables simultaneously, followed by Tukey’s *posthoc* analysis for multiple group comparisons. Vascular reactivity data were analyzed by nonlinear regression analysis (curve fit) and significance of differences was determined by comparing two curves for the fitted line. Results were considered significant if p-values were ≤0.05.

## Data Availability

Source data files are provided.

## Additional files

Supplementary file 1. Data details for graphs presented in the manuscript.

MDAR checklist
