# Supplementary material for: p66Shc mediates SUMO2-induced endothelial dysfunction
Source: eLife. 2026 Oct 21;14:RP106344. doi: 10.7554/eLife.106344 (PMC13686634; doi:10.7554/eLife.106344)

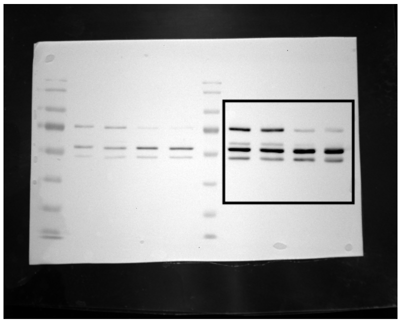

Supplement: Supplementary file 2 [file elife-106344-fig1-data1.zip › Figure 1-Source Data 1. PDF file containing original western blots for Figure 1A/ib_shc_ros_042523_b3p110_sk_final_(Chemi).tif.pdf]
