# Supplementary material for: p66Shc mediates SUMO2-induced endothelial dysfunction
Source: eLife. 2026 Oct 21;14:RP106344. doi: 10.7554/eLife.106344 (PMC13686634; doi:10.7554/eLife.106344)

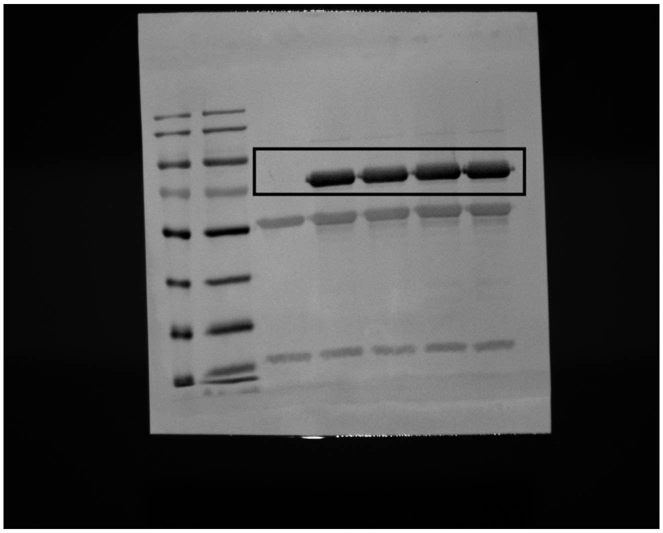

Supplement: Supplementary file 6 [file elife-106344-fig2-figsupp1-data1.zip › Figure 2-figure supplement 1-Source Data 1. PDF file containing original western blots for Figure 2-figure supplement 1/ip_flag_ib_flag_p66_sumo3_040223_b3p101r_(1_IRDye 680).tif.pdf]
