# Supplementary material for: p66Shc mediates SUMO2-induced endothelial dysfunction
Source: eLife. 2026 Oct 21;14:RP106344. doi: 10.7554/eLife.106344 (PMC13686634; doi:10.7554/eLife.106344)

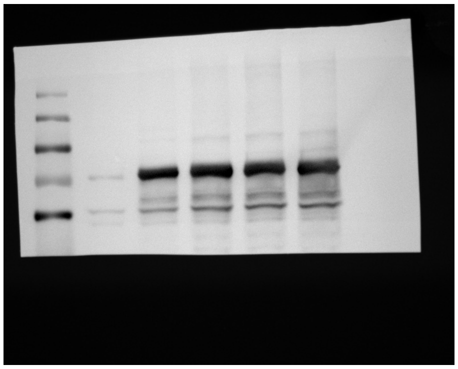

Supplement: Supplementary file 10 [file elife-106344-fig2-figsupp2-data3.zip › Figure 2-figure supplement 2B-Source Data 1. PDF file containing original western blots for Figure 2-figure supplement 2B/ib_shc_aa_b3p107_5324_(1_IRDye 680).jpg.pdf]
