# Supplementary material for: p66Shc mediates SUMO2-induced endothelial dysfunction
Source: eLife. 2026 Oct 21;14:RP106344. doi: 10.7554/eLife.106344 (PMC13686634; doi:10.7554/eLife.106344)

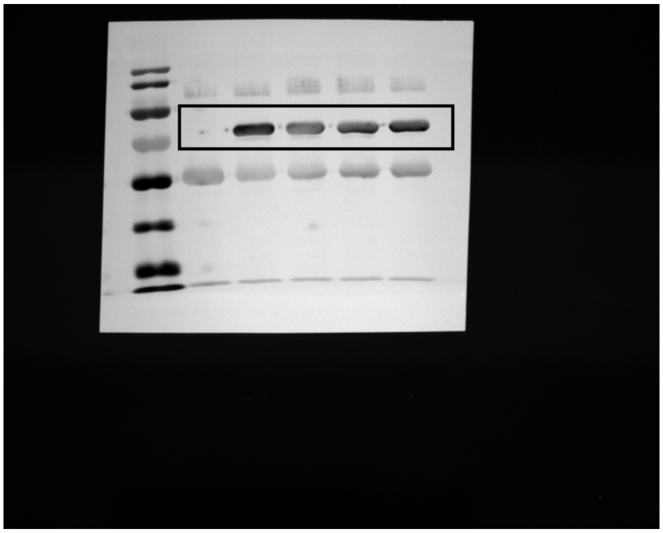

Supplement: Supplementary file 12 [file elife-106344-fig3-data1.zip › Figure 3-Source Data 1. PDF file containing original western blots for Figure 3C/IP_FLAG_IB_FLAG_P66_K81R_08142021_B1_P79_(1_IRDye 680).tif.pdf]
