# Supplementary material for: p66Shc mediates SUMO2-induced endothelial dysfunction
Source: eLife. 2026 Oct 21;14:RP106344. doi: 10.7554/eLife.106344 (PMC13686634; doi:10.7554/eLife.106344)

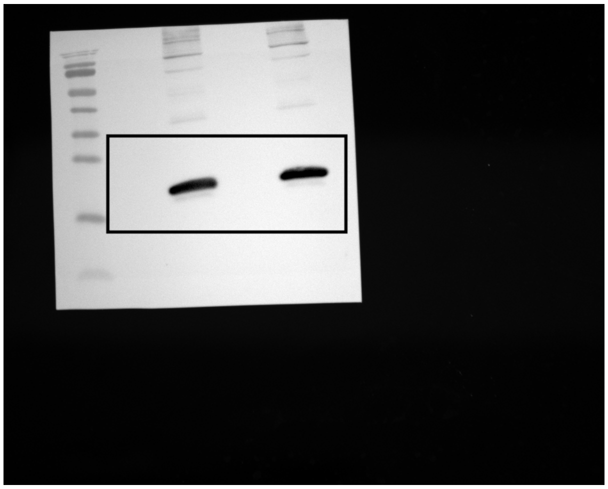

Supplement: Supplementary file 14 [file elife-106344-fig3-data3.zip › Figure 3-Source Data 1. PDF file containing original western blots for Figure 3D/IB_HA_P66_K81R_HUVEC_10182021_B1_P95_(1_IRDye 800).tif.pdf]
