# Supplementary material for: p66Shc mediates SUMO2-induced endothelial dysfunction
Source: eLife. 2026 Oct 21;14:RP106344. doi: 10.7554/eLife.106344 (PMC13686634; doi:10.7554/eLife.106344)

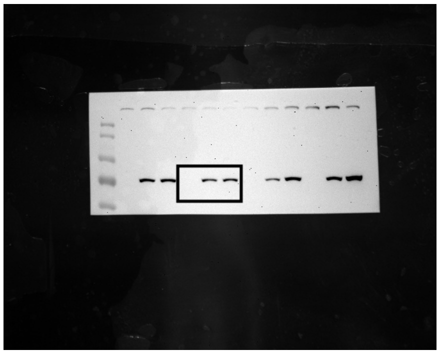

Supplement: Supplementary file 23 [file elife-106344-fig4-data2.zip › Figure 4-Source Data 1. PDF file containing original western blots for Figure 4A/ib_ps36_ps36_022623_b3p85r_(Chemi).jpg.pdf]
