# Supplementary material for: p66Shc mediates SUMO2-induced endothelial dysfunction
Source: eLife. 2026 Oct 21;14:RP106344. doi: 10.7554/eLife.106344 (PMC13686634; doi:10.7554/eLife.106344)

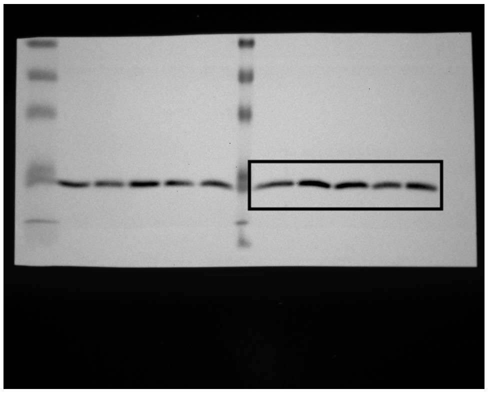

Supplement: Supplementary file 28 [file elife-106344-fig5-data1.zip › Figure 5-Source Data 1. PDF file containing original western blots for Figure 5A/ib_cox4_mit_loc_41523_b3p111_g2_(1_IRDye 800).tif.pdf]
