# Supplementary material for: p66Shc mediates SUMO2-induced endothelial dysfunction
Source: eLife. 2026 Oct 21;14:RP106344. doi: 10.7554/eLife.106344 (PMC13686634; doi:10.7554/eLife.106344)

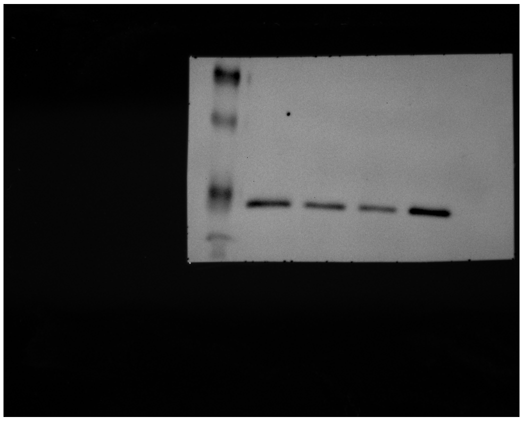

Supplement: Supplementary file 30 [file elife-106344-fig5-data3.zip › Figure 5-Source Data 1. PDF file containing original western blots for Figure 5C/ib_cox4_mit_frac_b3p69_12922_g2_2_(1_IRDye 800).tif.pdf]
