# Supplementary material for: p66Shc mediates SUMO2-induced endothelial dysfunction
Source: eLife. 2026 Oct 21;14:RP106344. doi: 10.7554/eLife.106344 (PMC13686634; doi:10.7554/eLife.106344)

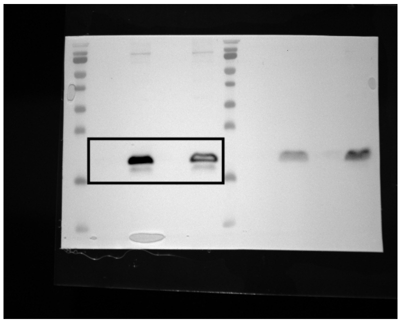

Supplement: Supplementary file 30 [file elife-106344-fig5-data3.zip › Figure 5-Source Data 1. PDF file containing original western blots for Figure 5C/IB_HA_MIT_LOC_6MAY22_B3P17_R2.tif.pdf]
