# Supplementary material for: p66Shc mediates SUMO2-induced endothelial dysfunction
Source: eLife. 2026 Oct 21;14:RP106344. doi: 10.7554/eLife.106344 (PMC13686634; doi:10.7554/eLife.106344)

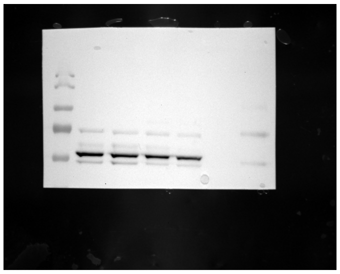

Supplement: Supplementary file 38 [file elife-106344-fig6-figsupp1-data1.zip › Figure 6-figure supplement 1A-Source Data 1. PDF file containing original western blots for Figure 6-figure supplement 1A/IB_Shcp66_08202023_163630_F_(Chemi).tif.pdf]
