# Supplementary material for: p66Shc mediates SUMO2-induced endothelial dysfunction
Source: eLife. 2026 Oct 21;14:RP106344. doi: 10.7554/eLife.106344 (PMC13686634; doi:10.7554/eLife.106344)

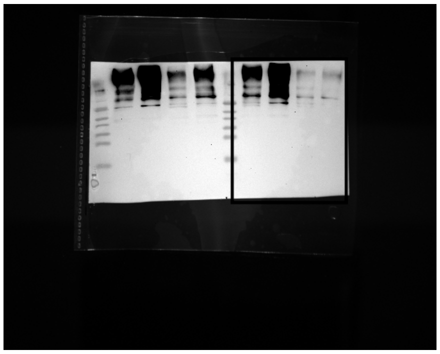

Supplement: Supplementary file 40 [file elife-106344-fig7-data1.zip › Figure 7-Source Data 1. PDF file containing original western blots for Figure 7A/IB_SUMO23_1_2_OLDL_HUVEC_11192021_B1_P112.tif.pdf]
