# Supplementary material for: p66Shc mediates SUMO2-induced endothelial dysfunction
Source: eLife. 2026 Oct 21;14:RP106344. doi: 10.7554/eLife.106344 (PMC13686634; doi:10.7554/eLife.106344)

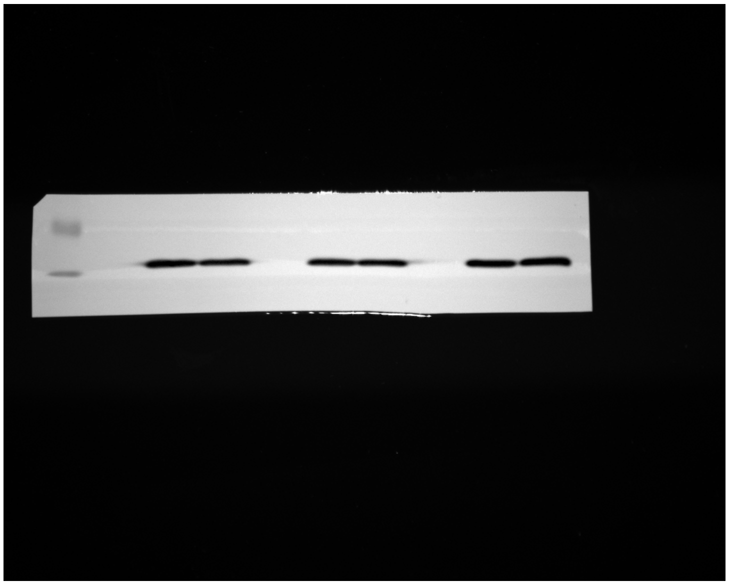

Supplement: Supplementary file 42 [file elife-106344-fig8-figsupp1-data1.zip › Figure 8-figure supplement 1A-Source Data 1. PDF file containing original western blots for Figure 4-figure supplement 1A copy/IB_HA_11092023_195927_11623_(1_IRDye 800).tif.pdf]
