# Supplementary material for: p66Shc mediates SUMO2-induced endothelial dysfunction
Source: eLife. 2026 Oct 21;14:RP106344. doi: 10.7554/eLife.106344 (PMC13686634; doi:10.7554/eLife.106344)
Supplement: Peer review history

# Editor report: eLife Assessment

**DOI:** 10.7554/eLife.106344.3.sa0

**Authors:** Ilse S Daehn

**Affiliations:** Icahn School of Medicine at Mount Sinai United States

**Keywords:** Compelling, Solid, Valuable

This study offers **valuable** insights into the role of post-translational modifiers, specifically SUMO2ylation at K81 in p66Shc, and its impact on endothelial function through reactive oxygen species. A series of **compelling** experiments demonstrated that lysine 81 of p66Shc is the site of SUMO2 conjugation, which is crucial for mitochondrial localization and essential for S36 phosphorylation, leading to specific pathological effects. The combination of cell overexpression and animal studies provides **solid** data supporting this mechanistic link.

# Referee report: Reviewer #2 (Public review)

**DOI:** 10.7554/eLife.106344.3.sa1

Summary:

The manuscript titled "p66Shc Mediates SUMO2-induced Endothelial Dysfunction" by Kumar et al. builds upon established literature demonstrating that both p66Shc and SUMOylation are essential players in nitric oxide (NO)-mediated endothelial vascular homeostasis and development (PMID: 10580504, 28760777, and 35187108).

In this study, the authors uncover a novel mechanism showing how the SUMO2ylation of p66Shc drives reactive oxygen species (ROS) production in endothelial cells. Specifically, they identify Lysine 81 (K81) as the critical residue on p66Shc conjugated to SUMO2, proving it is essential for the protein's mitochondrial localization.

The authors convincingly demonstrate that:

p66Shc is actively SUMO2ylated at the K81 site in cellular models.

Phosphorylation at Serine 36 (S36) is significantly reduced upon the loss of this critical SUMOylation site.

Conclusion:

Overall, this study provides strong evidence for a novel regulatory axis in endothelial cells. It successfully opens the door for further dissection of the complex mechanistic crosstalk between three key post-translational modifications on p66Shc: S36 phosphorylation, K81 SUMO2ylation, and acetylation.

# Author comment: Author response

**DOI:** 10.7554/eLife.106344.3.sa2

**Authors:** Jitendra Kumar, Shravan K Uppulapu, Sujata Kumari, Kanika Sharma, William J Paradee, Ravi Prakash Yadav, Vikas Kumar, Santosh Kumar

**Affiliations:** University of Iowa Iowa City United States; University of Iowa Iowa City United States; University of Iowa Iowa City United States; University of Nebraska Medical Center Omaha United States; University of Iowa Iowa City United States; Regional Institute of Education-NCERT Mysore India; University of Massachusetts Worcester United States; University of Iowa Iowa City United States

The following is the authors’ response to the original reviews.

**Public Reviews:**

**Reviewer #1 (Public review):**

Summary:

The authors describe a role of sumoylation at K81 in p66Shc which affects endothelial dysfunction. This explores a new mechanism for understanding the role of PTMs in cellular processes.

Strengths:

The experiments are well planned and the results are well represented.

Vascular tonality experiments were carried out nicely, given the amount of time and effort one needs to put in to get clean results from these experiments.

Weaknesses:

(1) The production of ROS has been measured in a very superficial way.

The term "ROS" confers a plethora of chemical species which exerts different physiological effects on different cells and situations.

Mitochondria through one of the source, but not the only source of ROS production. Only measuring ROS with mitosox do not reflect the cellular condition of ROS in a specific condition. I would suggest authors consider doing IF of oxidative stress specific markers, carbonyl group and also, maybe, Amplex red for determining average oxidative stress and ros production in the cells.

As suggested, we employed an additional ROS-sensitive probe, H_2_DCFDA, which revealed an overall increase in intracellular ROS levels upon SUMO2 overexpression; this effect was reversed by knockdown of p66Shc. In addition, we performed the Amplex Red assay on conditioned media to assess extracellular ROS release. SUMO2 overexpression did not alter ROS levels detected in the media, whereas a paradoxical increase was observed following p66Shc knockdown.

**Author response image 1.** Amplex Red assay performed in HUVECs with and without knockdown of p66Shc expressing SUMO2 (Ad-SUMO2) or a control virus (Ad-LacZ).

Amplex Red predominantly detects hydrogen peroxide (H_2_O_2_), which may originate from NADPH oxidases or be generated through the dismutation of superoxide. In contrast to superoxide, H₂O₂ is relatively stable, membrane-permeable, and well recognized as a second messenger in endothelial signaling, where it modulates kinase and phosphatase activity and supports physiological vascular functions. Thus, the elevated H_2_O_2_ detected following p66Shc knockdown may represent a signaling-competent redox state rather than a pathological increase in oxidative stress. Moreover, the SUMO2-p66Shc-mediated increase in mitochondrial ROS may be efficiently buffered by cellular antioxidant defense mechanisms, thereby limiting detectable extracellular ROS release.

(2) 8-OHG signal seems very confusing in Figure 7E. 8-ohg is supposed to be mainly in the nucleus and to some extent in mitochondria. The signal is very diffused in the images. I would suggest a higher magnification and better resolution images for 8-ohg. Also, the VWF signal is pretty weak whereas it should be strong given the staining is in aorta. Authors should redo the experiments.

We have provided a better image for figure 7E. We repeated the staining with another antibody for vWF which showed stronger signal for vWF.

(3) PCA analysis is quite not clear. Why is there a convergence among the plots? Authors should explain. Also, I would suggest that the authors do the analysis done in Figure 8B again with R based packages. IPA, though being user-friendly, mostly does not yield meaningful results and the statistics carried out is not accurate. Authors should redo the analysis in R or Python whichever is suitable for them.

We thank the reviewer for their valuable feedback and insightful suggestions.

Regarding the PCA analysis, the observed convergence among the data points reflects the underlying biological similarity between the samples within each group. Given the relatively low abundance and limited number of quantified peptides in our dataset, the variance captured by PCA is modest, leading to partial overlap between groups. This convergence is therefore likely due to shared biological characteristics and inherent sample variability, rather than technical issues.

In response to the reviewer’s suggestion on pathway analysis, we have re-performed the analysis using R-based approaches. Specifically, we utilized established pipelines PROGENy-based signaling pathway activity inference. These methods provide statistically robust and reproducible results. The updated analyses and corresponding figures have been included in the revised manuscript, replacing the previous IPA-based results (Fig. 8). We believe these additions strengthen the interpretation of signaling pathway alterations in our dataset.

(4) The MS analysis part seems pretty vague in methods. Please rewrite.

We have revised the methodology for MS.

**Reviewer #2 (Public review):**

Summary:

The article builds on the earlier work that both p66Shc and SUMOylation are essential nitric oxide (NO) based development of endothelial vasculature (PMID: 10580504; 28760777 and 35187108). The current manuscript brings forward a finding of how SUMO2ylation of p66Shc mediated ROS production which is essential for endothelial cells. They further identify that lysine 81 of p66Shc is the residue which is conjugated to SUMO2 and is crucial for mitochondrial localization. They further show that K81 SUMO2ylation is essential for S36 phosphorylation.

Strengths:

Convincingly shows that p66Shc is SUMO2ylated on lysine 81 in cells and also shows that the phosphorylation (serine 36) reduces upon loss of this critical SUMOylation site.

Weaknesses:

All the experiments performed here are in overexpression background therefore, it would be crucial to show that p66Shc is SUMO2ylated at physiological levels.

As detecting endogenous SUMO2-p66Shc is technically challenging considering the almost 92% homology between SUMO2 and SUMO3 and the absence of a specific antibody to detect p66Shc, we generated a custom-made antibody which can detect SUMO2-p66Shc (YenZym, CA). Using this antibody, we performed immunoprecipitation which showed endogenous SUMO2-p66Shc at a molecular weight higher than p66Shc suggesting the SUMO2 modification of p66Shc at physiological level.

**Reviewer #3 (Public review):**

Summary:

The authors set out to determine how SUMO2 impairs endothelial function through direct modification of the protein p66Shc. p66Shc is known to promote reactive oxygen species production, and here the authors demonstrate that SUMO2 modifies p66Shc at lysine-81, resulting in increased phosphorylation, mitochondrial translocation. These are prosed to mediate the detrimental effects of SUMO2 in a mouse model of hyperlipidemia.

Strengths:

A major strength of this work is the multi-pronged approach combining biochemical assays, proteomic analyses, and a genetically modified mouse model expressing a SUMOylation resistant mutant of p66Shc. These experiments comprehensively illustrate that lysine-81 SUMOylation of p66Shc is necessary for the observed endothelial dysfunction in hyperlipidemic conditions.

Weaknesses:

One notable weakness is that the link between the observed cellular changes and the ultimate in vivo phenotype remains only partially explored. While the authors successfully show that p66ShcK81R knockin mice are protected from endothelial dysfunction in a hyperlipidemic context, additional experiments characterizing the broader tissue-specific roles, or examining further endothelial assays in vivo, would strengthen the mechanistic conclusions. It would also be beneficial to see more direct evaluations of p66Shc subcellular localization in the protective knockin mice to complement the proteomic findings.

We agree with the reviewer’s suggestion. However, due to limited resources, we could not pursue additional studies.

Despite these gaps, the data broadly support the authors' main conclusions. The authors lay out a plausible mechanistic pathway for how hyperlipidemia and increased global SUMOylation can converge on the oxidative stress pathway to provoke vascular dysfunction.

The likely impact of this work on the field is noteworthy. Beyond clarifying how a single post-translational modification event can influence the pathophysiology of endothelial cells, the study provides a model for investigating broader roles of SUMO2 in other cardiovascular conditions and highlights the importance of identifying additional SUMOylation sites and their downstream impact.

In conclusion, by demonstrating the direct SUMOylation of p66Shc at lysine-81 and linking that modification to endothelial dysfunction in a hyperlipidemic mouse model, this paper offers valuable insights into how broadly acting post-translational modifiers can evoke specific pathological effects.

**Recommendations for the authors:**

**Reviewer #1 (Recommendations for the authors):**

(1) Please rearrange the figures. It is very hard to follow.

We rearranged some of the figures for a better presentation.

**Reviewer #2 (Recommendations for the authors):**

**Please note that I am not an expert of mouse work therefore I will not be able to comment on the mouse work (Figure 7).

The following suggested changes major concerns will strengthen the work, and the minor concerns will increase the paper's accessibility to eLife's broad scientific community.

Major concerns.

(1) All the work done here is based on overexpression studies, therefore it will be very useful to show that p66Shc gets SUMO2ylated under physiological conditions using SUMO-Trap beads or using tandem SUMO-Interacting Motifs (as shown in Silva-Ferrada et al., 2013: https://doi.org/10.1038/srep01690).

We have demonstrated endogenous SUMO2 conjugation of p66Shc under physiological conditions by immunoprecipitation using a custom-generated antibody specific for SUMO2-p66Shc. This approach allows detection of SUMO2ylated p66Shc without reliance on overexpression systems, thereby confirming that SUMO2 modification of p66Shc occurs endogenously.

(2) The authors claim that K81 is the only site of SUMO2ylation and based on the HUVEC experiments (Fig 3C, D) it appears that p66Shc K81R mutant still gets SUMO2ylated indicating that there are other residues which can get SUMO2ylated. Do you see the other sites getting SUMO2ylated in your mass spec data?

Our mass spectrometry analysis did not identify SUMO2 modification on lysine residues other than K81. However, we acknowledge that SUMOylation detected in vitro on recombinant protein may differ from SUMOylation occurring in a cellular context, where protein conformation, interacting partners, and local enzyme availability can influence modification patterns. These differences may account for the appearance of multiple SUMOylated p66Shc species in cell lysates despite the absence of additional SUMOylation sites in the mass spectrometry dataset. Our emphasis on K81 is based on its localization within the CH2 domain of p66Shc, a region unique to the p66 isoform. Previous studies have demonstrated that post-translational modifications within the CH2 domain, such as phosphorylation, are critical for activation of the oxidative and pro-apoptotic functions of p66Shc. Accordingly, we focused our mechanistic analyses on SUMO2ylation at K81. Nevertheless, we do not exclude the possibility that additional lysine residues within the PTB or CH1 domains of p66Shc may also undergo SUMOylation in cells. Importantly, our functional data support the conclusion that SUMO2ylation at K81 is a key regulatory modification driving the oxidative activity of p66Shc.

(3) In Figure 4 the authors claim that SUMO2ylation at K81 is a prerequisite for S36 phosphorylation, however the phosphorylation changes are marginal (Figure 4 A, E) and why do they not see the higher molecular weight bands in the western blots.

We appreciate the critique and agree with the reviewer that our data do not provide direct evidence that K81 SUMO2ylation and S36 phosphorylation occur simultaneously on the same p66Shc molecule. Rather, our findings suggest that SUMO2ylation at K81 may facilitate or enhance S36 phosphorylation, but is not an absolute requirement for this modification. Accordingly, we have revised the wording in the main text to avoid implying a strict prerequisite relationship and to more accurately reflect the magnitude of the observed changes in S36 phosphorylation.

(4) The authors further claim that the SUMO2ylation at K81 effects S36 phosphorylation which has consequences for mitochondrial localization. However, K81R still translocate to mitochondria which is indicative of SUMO2ylation is important but not necessary (Fig 5A, C).

We agree with the reviewer’s observation and have revised the main text accordingly, as described in our prior response, to clarify that K81 SUMO2ylation facilitates, but is not essential for, S36 phosphorylation and mitochondrial translocation.

(5) To know if the K81 SUMO2ylation had any effect on phosphorylation it would be interesting to see how the phosphomimic (S36D) mutant along with/without K18R will behave in mitochondrial localization experiments.

We agree that examining the double mutant (S36D/K81R) would provide valuable mechanistic insight into the relationship between K81 SUMO2ylation and S36 phosphorylation in regulating mitochondrial localization. Although we are currently unable to perform these experiments due to constraints in manpower and resources, we have acknowledged this as a limitation in the Discussion and plan to pursue these studies in future work to further validate the mechanism.

Minor Concerns

(a) In Figure 1A the authors claim that SUMO2ylation of p66Shc promotes ROS production however they do not include any well-known ROS induced proteins in the western blots such as KEAP1 or NRF2 or any other marker.

We agree that NRF2 is a well-established transcriptional regulator of antioxidant responses. However, the primary aim of Figure 1A was to directly examine the effect of SUMO2ylation on p66Shc-mediated ROS production. While NRF2 and other ROS-responsive proteins reflect downstream adaptive responses, they do not provide a direct measure of ROS generation. Therefore, we focused on measuring SUMO2-induced changes in cellular ROS levels. To further strengthen this conclusion, we have performed additional complementary ROS assays, which are now included in the revised manuscript.

(b) In Figure 2D, the concentration of Anacardic acid used is low and therefore the SUMO2ylation is not completely inhibited. It would be advisable if the authors go to concentration of complete inhibition.

We acknowledge that some residual SUMO2ylation is visible in the immunoblots following anacardic acid treatment. However, the assay demonstrates a clear, dose-dependent reduction in SUMO2ylation levels, which is sufficient to interpret the effect of SUMO2 inhibition on p66Shc function. Increasing the concentration further could introduce off-target effects, and the current assay provides a reliable and physiologically relevant readout.

(c) The figure panels need uniform fonts and also the size/resolution of the western blots needs to be improved

We thank the reviewer for this suggestion. All figures have been updated to ensure uniform fonts, and the western blot images have been improved for size and resolution in the revised manuscript.

(d) Supplemental figures are very low resolution.

High-resolution images have been provided.

(e) Please introduce abbreviations before using them (like LDLr and ND).

The abbreviations have been explained.

(f) Figure 6A seems like data that belongs in the SI rather than the main text.

We kept it in main figure as the knock-in mouse was generated for this study.

(a) Figure 7B needs a WT HFD control.

It is a valid suggestion. However, it is known in the literature (we have prior experience as well) that wild-type mice do not exhibit a drastic increase in serum lipid level with high-fat diet feeding.

(b) The differences in Figure 7E are not profound and immediately obvious. Is there a better way to show the data, potentially with quantification?

We agree, the difference is not that huge, we have provided the qualification as Fig. 7F.

(h) Figure panels 6E-H could use some labels to differentiate the data from WT vs p66ShcK81R mutant mice within the figure panel.

We mentioned that on the top and have inserted a vertical line to separate the groups.

(i) Lines 165-168, 182-185, 214-218 and 282-286: These sentences can be rewritten for more clarity.

We have modified these sentence for clarity.

(j) Line 512 should read, "Two-way ANOVA, ***P<0.001."

Thank you, we have corrected the mistake.
